# Implications of Individual QT/RR Profiles—Part 1: Inaccuracies and Problems of Population-Specific QT/Heart Rate Corrections

**DOI:** 10.1007/s40264-018-0736-1

**Published:** 2018-09-25

**Authors:** Marek Malik, Christine Garnett, Katerina Hnatkova, Jose Vicente, Lars Johannesen, Norman Stockbridge

**Affiliations:** 1 National Heart and Lung Institute, Imperial College, Dovehouse Street, London, SW3 6LY England, UK; 2 Division of Cardiovascular and Renal Products, Office of New Drugs, Center for Drug Evaluation and Research, US Food and Drug Administration, Silver Spring, MD USA; 3 Division of Clinical Pharmacology I, Office of Clinical Pharmacology, Center for Drug Evaluation and Research, US Food and Drug Administration, Silver Spring, MD USA

## Abstract

**Introduction:**

Universal QT correction formulas are potentially problematic in corrected QT (QTc) interval comparisons at different heart rates. Instead of individual-specific corrections, population-specific corrections are occasionally used based on QT/RR data pooled from all study subjects.

**Objective:**

To investigate the performance of individual-specific and population-specific corrections, a statistical modeling study was performed using QT/RR data of 523 healthy subjects.

**Methods:**

In each subject, full drug-free QT/RR profiles were available, characterized using non-linear regression models. In each subject, 50 baseline QT/RR readings represented baseline data of standard QT studies. Using these data, linear and log-linear heart rate corrections were optimized for each subject and for different groups of ten and 50 subjects. These corrections were applied in random combinations of heart rate changes between − 10 and + 25 beats per minute (bpm) and known QTc interval changes between − 25 and + 25 ms.

**Results:**

Both the subject-specific and population-specific corrections based on the 50 baseline QT/RR readings tended to underestimate/overestimate the QTc interval changes when heart rate was increasing/decreasing, respectively. The result spread was much wider with population-specific corrections, making the estimates of QTc interval changes practically unpredictable.

**Conclusion:**

Subject-specific heart rate corrections based on limited baseline drug-free data may lead to inconsistent results and, in the presence of underlying heart rate changes, may potentially underestimate or overestimate QTc interval changes. The population-specific corrections lead to results that are much more influenced by the combination of individual QT/RR patterns than by the actual QTc interval changes. Subject-specific heart rate corrections based on full profiles derived from drug-free baseline recordings with wide QT/RR distribution should be used when studying drugs expected to cause heart rate changes.

**Electronic supplementary material:**

The online version of this article (10.1007/s40264-018-0736-1) contains supplementary material, which is available to authorized users.

## Key Points

**Table:** 

In the presence of non-trivial drug-induced heart rate changes, population-specific corrections lead to estimates of corrected QT (QTc) interval changes that are biased in a way that is impossible to predict. Consequently, population-specific corrections should not be used.
Estimates of QTc interval changes based on subject-specific corrections derived from narrow windows of baseline data may also lead to inconsistent results.
Full intra-subject profiles of the baseline QT/RR relationship over wide heart rate ranges are needed to design subject-specific corrections that may be relied on in investigations of drugs that change the heart rate considerably.

## Introduction

In studies of drug-induced corrected QT (QTc) interval changes [1], the accuracy of heart rate correction of the QT interval becomes particularly important when the investigated drug changes the heart rate [2]. The same applies to other QTc interval studies, e.g., analyses of QTc interval changes due to provocative maneuvers, instrumental interventions, and disease progression.

The guidance document by Cardiac Safety Research Consortium [2] explains that in situations of appreciable heart rate changes, fixed universal heart rate corrections are not necessarily reliable and subject-specific heart rate corrections need to be used. The document also suggests that the appropriateness of subject-specific heart rate corrections depends on the wide range of drug-free QT and RR measurements in each individual. Collecting such a wide range of drug-free QT/RR pairs brings challenges to the study design, e.g., when incorporating the assessment of QTc interval changes into early clinical studies [3]. In addition, it is especially challenging obtaining QT/RR pairs at low heart rates, e.g., in QT studies assessing a drug that is expected to reduce the heart rate.

Consequently, suggestions have occasionally been made to use heart rate corrections specifically designed not for each study subject separately but based on baseline drug-free QT/RR data pooled from all study participants. It is assumed that such a population-specific correction addresses the accuracy of QTc interval data reasonably, but little data exist on the appropriateness of these corrections [4].

To address this lack of systematic data on the appropriateness of population-specific correction, we have designed and performed a statistical modeling study that utilized a large set of data from previously conducted thorough QT investigations. As a by-product of this study, we have also assessed the appropriateness of subject-specific corrections utilizing limited drug-free QT/RR datasets in separate subjects.

## Methods

### Subjects and Data

Data from two previous large thorough QT studies were available for this modeling study [5]. Together, the studies investigated 523 healthy subjects (254 females), with a mean age of 33.5 years and an interquartile range (IQR) of age of 26.6–40.1 years. Both source thorough QT studies were appropriately approved by regulatory and relevant ethics bodies, but since we used only their drug-free QT/RR data, their other details are not relevant. No subject participated in both source studies.

In each subject, multiple (average *n* = 1263, IQR 1060–1437) baseline drug-free daytime QT interval measurements were available together with corresponding QT/RR hysteresis corrected RR intervals [6] representing the underlying heart rate at which the QT interval was measured. These drug-free QT/RR measurements covered wide ranges of heart rate in each subject. The average minimum heart rate in these QT/RR measurements was 51.9 beats per minute (bpm) (IQR 47.8–56.1); the average maximum heart rate was 112.5 bpm (IQR 102.9–122.9).

The QT/RR hysteresis correction was based on individual models of exponential decay of the influence of RR intervals preceding the QT interval measurements. For each QT interval measurement, the corresponding RR interval was obtained as a weighted average of RR intervals within 5 min preceding the QT measurement.

The dense baseline QT/RR data distribution allowed us to describe the drug-free QT/RR relationship in each subject using the previously published curvilinear regression formula [7]:

$${\text{QT}} = {\text{QTcI}} + (\delta /\gamma )({\text{RR}}^{\gamma } - 1),$$

which corresponds to the correction formula:

$${\text{QTcI}} = {\text{QT}} + (\delta /\gamma )(1 - {\text{RR}}^{\gamma } ),$$

where QTcI is the individually corrected QT interval and individually optimized parameters *δ* and *γ* represent the slope and the curvature of the QT/RR relationship, respectively (QT and RR interval measurements are expressed in seconds).

In each subject, we used ten baseline datapoints distributed through the daytime hours. This modeled drug-free points during a QT investigation. The subjects were in supine resting positions for at least 5 min before as well as during these datapoints. The electrocardiogram (ECG) measurements of each of the selected datapoints consisted of five replicated QT and RR measurements (the RR values were again QT/RR hysteresis corrected). That is, the selected datapoints provided 50 QT and RR measurements per subject. The intra-subject spread of heart rates measured during these baseline datapoints corresponded to the usual data distribution seen in supine datapoints of clinical investigations of QT interval changes [6]. The average minimum and maximum heart rates of the datapoints were 55.5 bpm (IQR 50.1–60.0) and 75.8 bpm (IQR 69.3–81.8), respectively.

### Statistical Modeling Experiments

To model the situation in which restricted baseline data are available for the design of subject-specific and population-specific heart rate corrections, we considered two types of modeling experiments in which the corrections were derived from the selected baseline datapoints, i.e., in which 50 pairs of QT and RR measurements were available in each subject.

The experiments of the first type (Type 1) modeled the situations of standard QT studies with relatively uniform heart rate and QT interval changes over all datapoints. This approximated the investigations after multiple drug doses when the drug plasma concentrations (and, correspondingly, drug effects on heart rate and on the QT interval) do not change during the final dosing day. For each such experiment, we considered 50 participants randomly selected from the 523 healthy subjects whose data were available. This size reasonably modeled the usual thorough QT investigations [1].

**Fig. 1:**
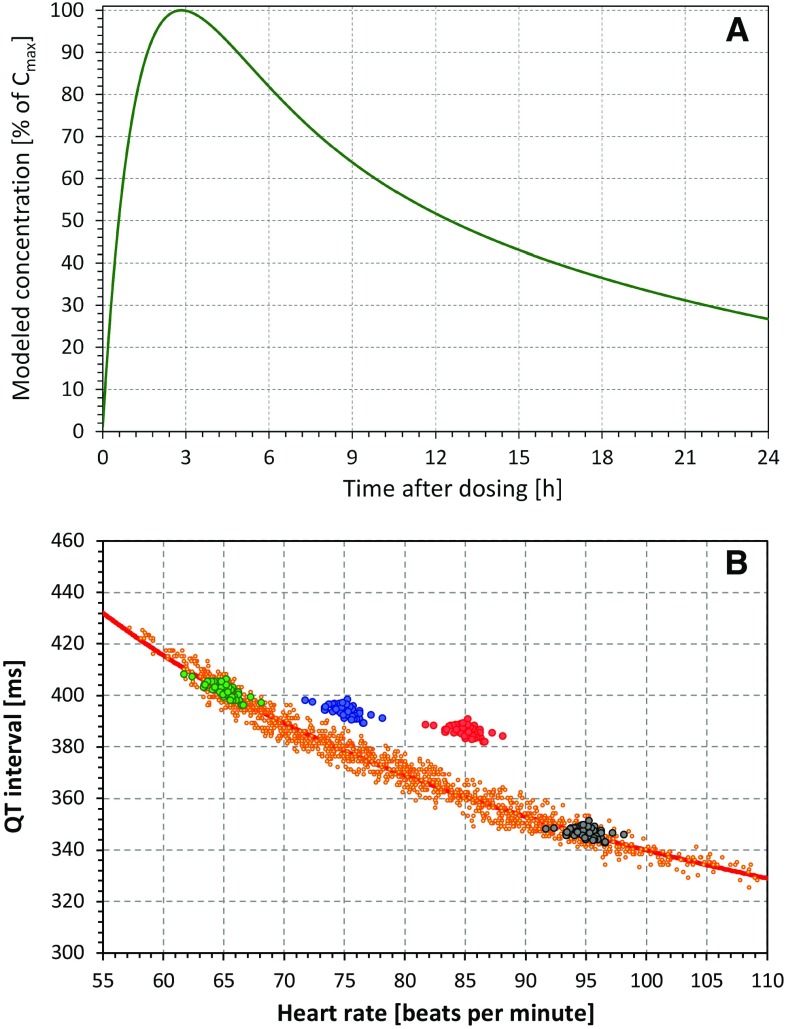
**a** Modeled plasma concentration profile used in the experiments of the second type (see text for details). **b** Schema of the modeled QT and heart rate changes. In each study subject, the full profile of the relationship between QT intervals and underlying heart rates (derived from hysteresis-corrected RR intervals) was obtained during previous investigations (small yellow dots). Their distribution allows curve-linear modeling of the QT/RR relationship (shown in the QT/heart rate plot as the solid red curvature). Replicated baseline measurements (green circles) coincide with the drug-free profiles. Their distribution and the curve-linear model of the QT/RR relationship allow simulation of different combinations of heart rate and QT changes. The figure shows three such simulations: heart rate increases of 10 bpm combined with QT increases of 15 ms are shown as blue circles; heart rate increases of 20 bpm combined with QT increases of 25 ms are shown as red circles; heart rate increases of 30 bpm with no QT increases are shown as black circles. Note that the black circles again coincide with the drug-free profile because no QT interval change was modeled. *C*_*max*_ maximum concentration

The experiments of the second type (Type 2) modeled situations in which only one drug dose is given to a relatively small number of individuals. In these experiments, we took the ten datapoints corresponding to 0, 1, 2, 3, 4, 5, 6, 8, 10, and 12 h after dosing and assumed that the averaged plasma concentrations (and, correspondingly, the heart rate and QT interval effects) followed the modeled concentration profile shown in Fig. 1a. For each of these experiments, we randomly selected ten participants, which approximately corresponded to the size of individual dose investigations in early clinical studies [3].

This means that in the experiments of the first type, the differences between the baseline and on-treatment QT/RR values were similar (subject to the modeled inaccuracy, as described further in Sect. 2.3), while in the experiments of the second type, the differences between the baseline and on-treatment data followed the modeled single-dose plasma concentration (i.e., ranged between no change at time 0 and maximum change at the point of maximum plasma concentration—again, subject to modeled inaccuracy). The experiments also differed in the number of subjects.

### QT/RR Changes

In all experiments, we modeled situations in which each subject also provided ten ‘on-treatment’ datapoints corresponding one-to-one to the selected baseline datapoints. Further, in each subject, the known values of averaged QTcI and of the coefficients *δ* and *γ* allowed us to estimate the true QT interval duration at any given heart rate. In individual experiments, we were therefore able to simulate situations when the on-treatment datapoints differed from the baseline datapoints by a prescribed amount. More specifically, for a selected datapoint measurement replicate with ECG baseline measurements of ${\text{RR}}_{b}$ and ${\text{QT}}_{b}$, we considered an on-treatment datapoint replicate with ECG measurements as follows:

$${\text{RR}}_{t} = 60/(60/{\text{RR}}_{b} + {\mathbb{C}}\varPhi_{\text{HR}} + \varepsilon_{\text{HR}} ),\,{\text{and}}$$

$${\text{QT}}_{t} = {\text{QT}}_{b} + (\delta /\gamma )({\text{RR}}_{t}^{\gamma } - {\text{RR}}_{b}^{\gamma } ) + {\mathbb{C}}\varPhi_{\text{QTc}} + \varepsilon_{\text{QTc}} ,$$

where $\varPhi_{\text{HR}}$ and $\varPhi_{\text{QTc}}$ were parameters of the experiment (modeling the drug-induced changes in the heart rate and of the QTc interval at 100% of maximum drug concentration), ${\mathbb{C}}$ was a drug concentration at the time of the given timepoint (expressed as a proportion to the maximum concentration), and $\varepsilon_{\text{HR}}$ and $\varepsilon_{\text{QTc}}$ were random inaccuracy coefficients. Since the modeling experiments need to operate on the uncorrected QT and RR values, the second formula was used for uncorrected ${\text{QT}}_{t}$ intervals. It was derived from the modeling assumption that:

$${\text{QTcI}}_{t} - {\text{QTcI}}_{b} = {\mathbb{C}}\varPhi_{\text{QTc}} + \varepsilon_{\text{QTc}} ,$$

from which the formula can be derived using the optimized correction:

$${\text{QTcI}} = {\text{QT}} + (\delta /\gamma )(1 - {\text{RR}}^{\gamma } ).$$

The principle of modeled QT and heart rate changes is shown schematically in Fig. 1b.

In the experiments of the first type, we assumed that all on-treatment measurements were influenced by the same maximum plasma concentration, whereas in the experiments of the second type we used the modeled plasma profile (Fig. 1a) and randomly assigned 10% variation of the plasma profile between individual subjects. The inaccuracy coefficients $\varepsilon_{\text{HR}}$ and $\varepsilon_{\text{QTc}}$ were introduced to model not only the measurement inaccuracies but also approximate the inter- and intra-subject differences and variability in the response as well as the differences between baseline and placebo (with no changes of heart rate or QT interval). Nevertheless, for the simplicity of interpretation of the results of the experiments, the $\varepsilon_{\text{HR}}$ and $\varepsilon_{\text{QTc}}$ coefficients were always obtained from uniformly distributed random numbers within ± 2 bpm and ± 5 ms, respectively.

### Heart Rate Corrections

In each modeling experiment, both subject-specific and population-specific heart rate corrections were considered. The subject-specific corrections considered the selected baseline datapoints of each subject separately; the population-specific corrections pooled the baseline datapoints of the subjects randomly selected for the experiment (i.e., of either 50 or ten subjects).

To reflect frequent practice, linear and log-linear correction formulas were derived. The linear correction formulas had the form of QTc = QT + *α*(1 − RR) and were derived from linear regressions QT = *α*_0_ + *α*RR; the log-linear correction formulas had the form of QTc = QT/RR^*β*^ and were derived from linear regressions log(QT) = *β*_0_ + *β*log(RR).

This means that in each modeling experiment, once the group of subjects was randomly selected and the coefficients *α* and *β* were obtained for each subject separately and subsequently, another pair of coefficients *α* and *β* was obtained for the group of the experiment pooled together.

To assess the impact of using limited versus full baseline QT/RR data, individualized corrections in the form ${\text{QTcI}} = {\text{QT}} + (\delta /\gamma )(1 - {\text{RR}}^{\gamma } )$ with the parameters optimized for each subject were also derived in the experiments.

### Organization of Experiments and Statistics

Experiments of both types were conducted for different combinations of programmed heart rate and QT interval changes. The parameters $\varPhi_{\text{HR}}$ and $\varPhi_{\text{QTc}}$ of heart rate and QTc interval changes were ranged systematically between − 10 and + 25 bpm in 0.1 bpm steps and between − 25 and + 25 ms in 0.1 ms steps, respectively. For each combination of the $\varPhi_{\text{HR}}$ and $\varPhi_{\text{QTc}}$ parameters, both types of experiment (i.e., the first type assuming the same heart rate and QTc interval changes at all selected timepoints, and the second type assuming changes according to the development of plasma concentrations) were repeated 50,000 times with different selections of modeled study populations. A Mersenne Twister random number generator was used [8].

In each individual experiment, the coefficient of subject-specific and population-specific heart rate corrections was optimized and applied to estimate the QTc interval changes between the baseline timepoints and corresponding on-treatment timepoints. In the experiments of the first type, the upper single-sided 95% confidence interval (CI) of the QTc interval changes was calculated for each of the timepoints and the resulting estimate of QTc interval changes was the maximum value of these upper CIs over all timepoints. This corresponded to the standard evaluation of thorough QT studies and resulted in the modeled estimates of the upper CI of $\Delta \Delta {\text{QTc}}$ values (i.e., QTc changes corrected for both baseline and placebo) expected for thorough QT studies [1]. In the experiments of the second type, the on-treatment timepoint of the maximum plasma concentration was used in each subject and in these timepoints, the upper single-sided 95% CI of the QTc interval changes was calculated. In both types of experiments, the upper CIs were calculated from values in individual subjects assuming normal distribution.

The repetition of modeling experiments led to 50,000 results (for experiments of both the first and second type) for each combination of programmed $\varPhi_{\text{HR}}$ and $\varPhi_{\text{QTc}}$ changes. Of these, the median value and the 5th, 10th, 20th, 80th, 90th, and 95th percentiles were obtained.

The principal outcomes of the study were the differences between the QTc interval changes reported by the investigated correction formulas and the initially programmed $\varPhi_{\text{QTc}}$ values. In ideal situations, the modeled $\Delta \Delta {\text{QTc}}$values should be the same as the initially programmed $\varPhi_{\text{QTc}}$ parameters of the experiments. The differences therefore showed how well or poorly the set-up of the investigated corrections represented the populations of the experiments. The relationships between these $\Delta \Delta {\text{QTc}} - \varPhi_{\text{QTc}}$inaccuracies and the programmed $\varPhi_{\text{HR}}$ heart rate changes were displayed graphically.

### Supplementary Analyses

To understand the reasons for inaccuracies in heart rate corrections, the linear and log-linear QT/RR slopes (i.e., the coefficients α and β as described previously) were compared when derived from full QT/RR profiles and from QT/RR data restricted to the selected baseline timepoints. The coefficients were compared using paired two-sided *t* tests. Where appropriate, data are presented as mean ± standard deviation.

Subsequently, in 100,000 randomly selected groups of ten and 50 subjects, the QT/RR data restricted to the selected baseline timepoints were used and population-specific linear and log-linear QT/RR slopes were compared with the averages of the subject-specific linear and log-linear slopes. That is, for each of these randomly selected groups, coefficients *α* and *β* were obtained for each subject as well as for the pool of baseline QT/RR data of all subjects together. The average of the individual coefficients was compared with the population coefficients.

## Results

### Source Data

The distribution of the QTcI values and of *δ* and *γ* parameters corresponded to the expectations of healthy subjects’ data [7], including the sex differences. In the total study population, the intra-individual regression residuals of the QTcI curvilinear regression models ${\text{QT}} + (\delta /\gamma )(1 - {\text{RR}}^{\gamma } )$ were 5.64 ± 1.12 ms.

While the averaged intra-subject differences between the fastest and slowest heart rates of the full QT/RR profiles were 60.7 bpm (IQR 50.5–70.4 bpm), the intra-subject range of heart rates of the selected datapoints was, on average, only 20.3 bpm (IQR 15.5–24.4 bpm). This difference in the spread of the of baseline data was mainly attributed to the missing values at accelerated heart rates, consistent with previous observations [6, 10].

### Differences Between Heart Rate Corrections

**Fig. 2:**
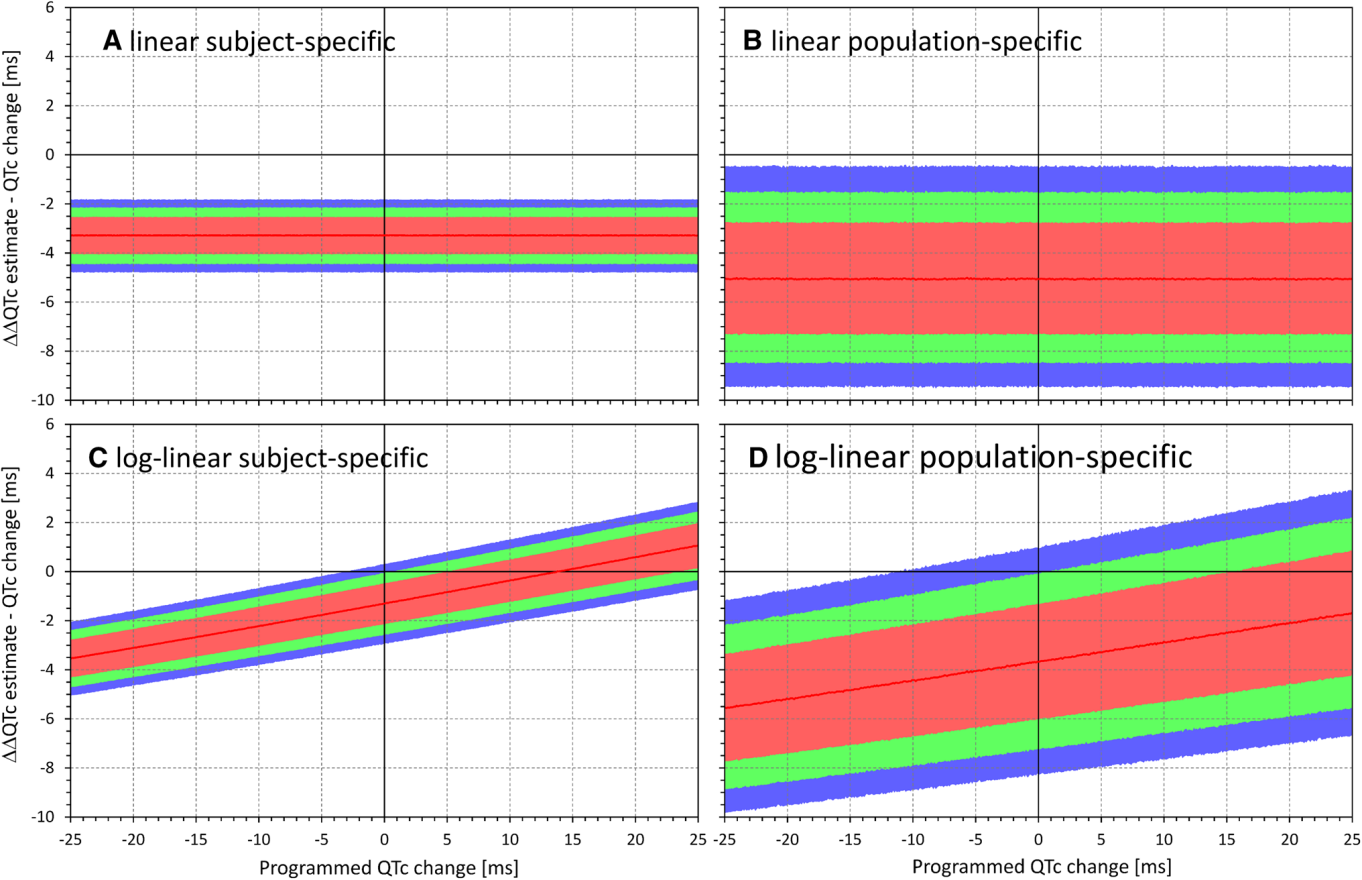
Example of experiments of the first type showing that the inaccuracies of heart rate corrections are practically independent of the programmed corrected QT (QTc) interval changes. In the experiments shown here, QTc changes programmed between − 25 and + 25 ms (horizontal axes) were all combined with heart rate acceleration of 15 beats per minute (bpm). The vertical axes of all panels show the errors in the QTc interval changes reported by the different correction formulas (reported minus actually programmed QTc interval change). Shown in this figure are linear heart rate correction optimized for individual subjects separately (**a**); linear heart rate correction optimized for study population (**b**); log-linear heart rate correction optimized for individual subjects separately (**c**); and log-linear heart rate correction optimized for study population (**d**). In all panels, the distribution of the correction errors in repeated experiment is shown (see text for details): the red lines show the median value and the pink, green, and blue bands show the ranges between 20th and 80th percentiles, 10th and 90th percentiles, and 5th and 95th percentiles, respectively

**Fig. 3:**
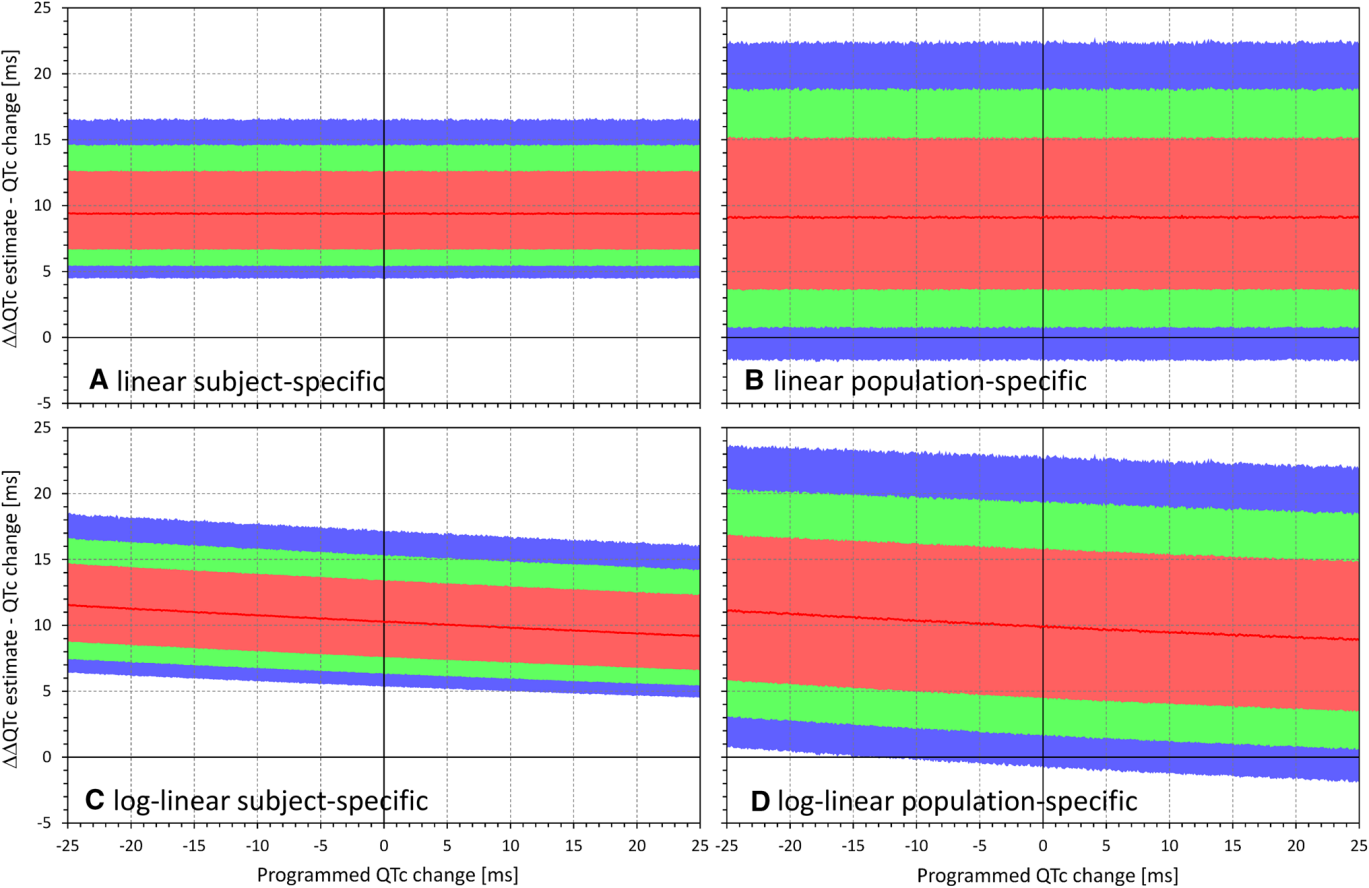
Example of experiments of the first type showing that the inaccuracies of heart rate corrections are practically independent of the programmed corrected QT (QTc) interval changes combined with heart rate deceleration of 10 beats per minute (bpm). Shown in this figure are linear heart rate correction optimized for individual subjects separately (**a**); linear heart rate correction optimized for study population (**b**); log-linear heart rate correction optimized for individual subjects separately (**c**); and log-linear heart rate correction optimized for study population (**d**). In all panels, the distribution of the correction errors in repeated experiment is shown (see text for details): the red lines show the median value and the pink, green, and blue bands show the ranges between 20th and 80th percentiles, 10th and 90th percentiles, and 5th and 95th percentiles, respectively. Compared with Fig. 2, it can be noted that (i) the spread of the results of the experiments modeling the corrections optimized for study population were wider than the experiments modeling the corrections optimized for individual subjects; and (ii) whilst the results of the linear corrections are truly independent on the setting of the QT changes, the heart rate accelerations and deceleration cause different slopes (in terms of QT change dependency) of the results of the log-linear corrections (because of the effects of the logarithmic transformation involved in the regression models)

Because of the linear additive properties [11], the inaccuracy of the linear corrections (both subject-specific and population-specific) are independent of the extent of the programmed $\varPhi_{\text{QTc}}$ changes (this is because with a linear correction, correcting QT intervals that differ by Δ for the same heart rate results in QTc interval values that again differ by Δ). This was not exactly the case with the log-linear corrections $\left( {{\text{because}}\,\frac{{{\text{QT}} + \Delta }}{{{\text{RR}}^{\beta } }} \ne \frac{\text{QT}}{{{\text{RR}}^{\beta } }} + \Delta } \right),$ but the dependency of their inaccuracies on the programmed $\varPhi_{\text{QTc}}$ changes was relatively modest. This is shown in Figs. 2 and 3; these figures show the results of the first type of experiments. Practically identical comparisons between subject-specific and population-specific corrections were observed with the experiments of the second type. Nevertheless, with the experiments of the second type, the bands of the inconsistency were much wider. For instance, with programmed heart rate changes of + 15 bpm and programmed QT interval changes of + 10 ms in the experiments of the second type, the width of the 90% CIs of the ΔΔQTc estimates was 7.2, 8.2, 22.2, and 24.1 ms for individual linear, individual log-linear, population linear, and population log-linear corrections, respectively.

The results in Figs. 2 and 3 show experiments that differed in the setting of QT changes $\varPhi_{\text{QTc}}$ but used the same setting of heart rate changes $\varPhi_{\text{HR}}$ of + 15 bpm (Fig. 2) and − 10 bpm (Fig. 3). These examples show that for linear heart rate corrections, both subject-specific and population-specific corrections tend to underestimate the QTc changes (ΔΔQTc values reported by the corrections were smaller than the programmed $\varPhi_{\text{QTc}}$ values) when the underlying heart rate was accelerated. On the contrary, the QTc interval changes were overestimated when heart rate was decelerated. The same holds true for log-linear corrections unless the heart rate accelerates substantially, which leads to the reversal of the effects.

**Fig. 4:**
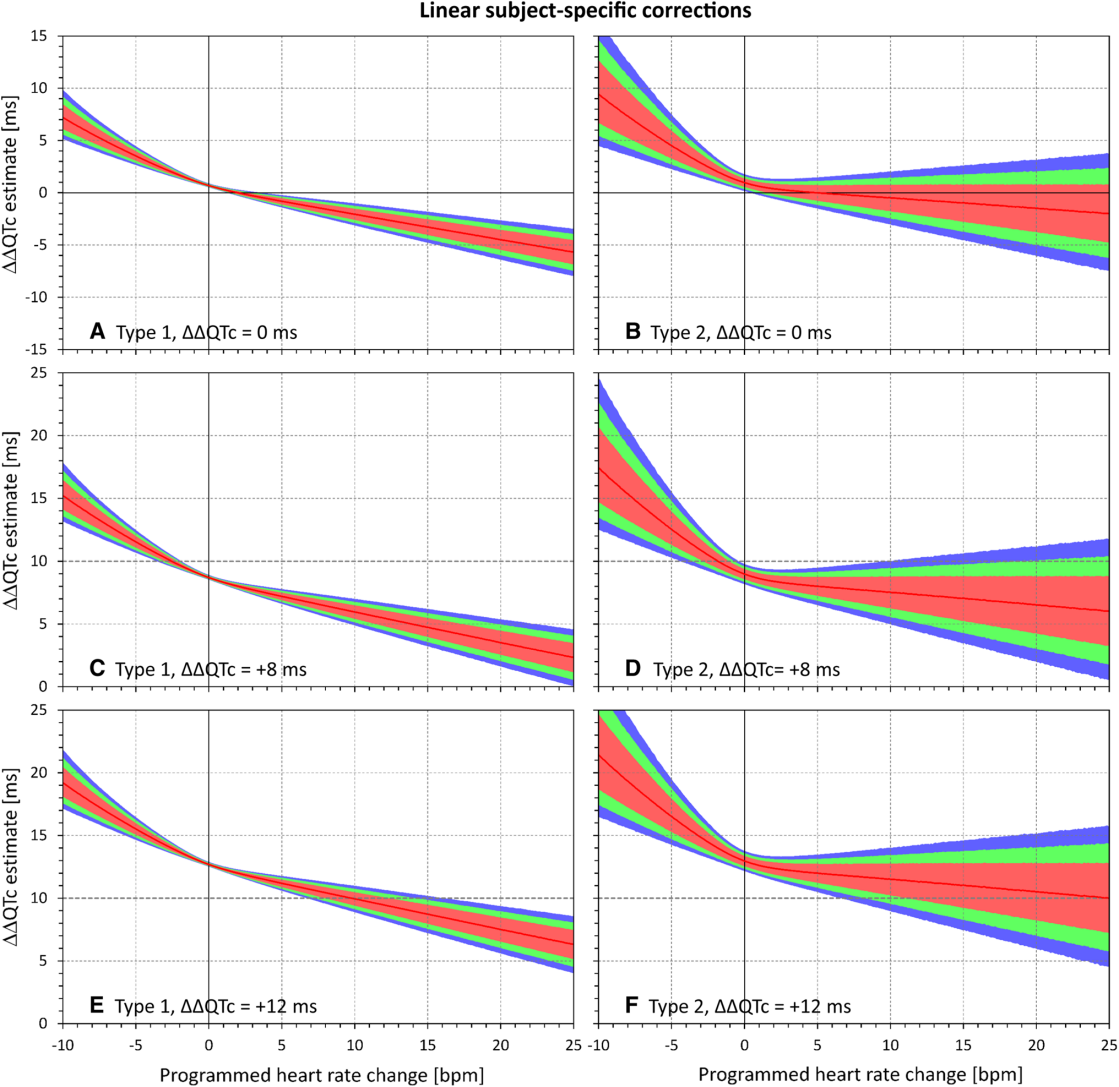
Dependency of the errors of investigated heart rate corrections on the programmed heart rate change. In the experiments shown, heart rate changes ranging from − 10 to + 25 beats per minute (bpm) were combined with programmed corrected QT (QTc) interval changes of 0 ms (**a**, **b**), + 8 ms (**c**, **d**), and + 12 ms (**e**, **f**). The graphs show the distribution of reported QTc interval changes obtained from linear corrections optimized for each subject separately. **a**, **c**, and **e** show the experiments of the first type (constant plasma concentrations in a larger group of subjects); **b**, **d**, and **f** show the experiments of the second type (variable plasma concentrations in a smaller group of subjects). In each panel, the distribution of the results in the repeated experiment is shown (see text for details): the red lines show the median value and the pink, green, and blue bands show the ranges between 20th and 80th percentiles, 10th and 90th percentiles, and 5th and 95th percentiles, respectively. The highlighted horizontal line in **c**–**f** shows the standard regulatory threshold of + 10 ms QTc interval change

**Fig. 5:**
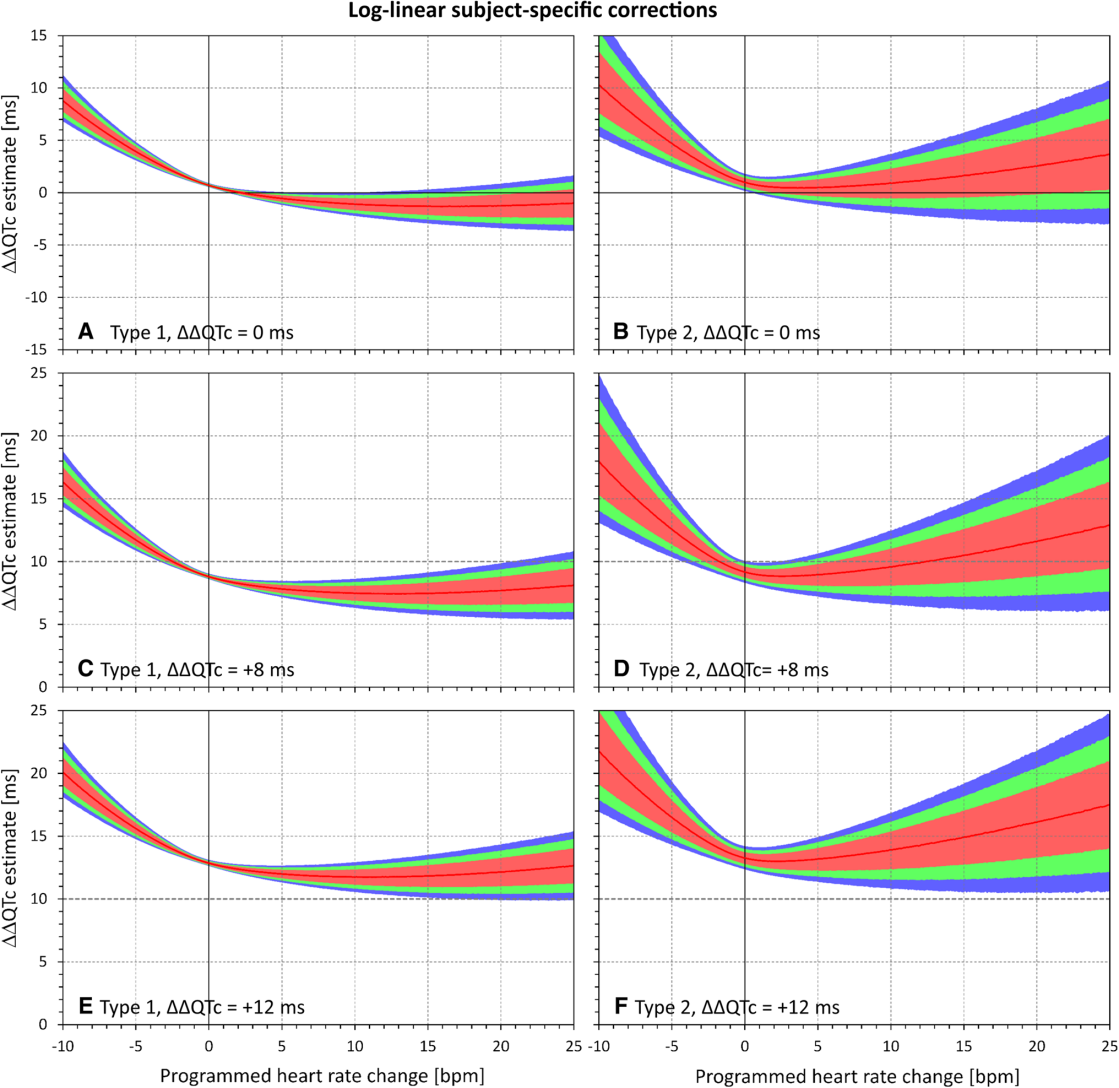
Dependency of the errors of investigated heart rate corrections on the programmed heart rate change. In the experiments shown, heart rate changes ranging from − 10 to + 25 beats per minute (bpm) were combined with programmed corrected QT (QTc) interval changes of 0 ms (**a**, **b**), + 8 ms (**c**, **d**), and + 12 ms (**e**, **f**). The graphs show the distribution of reported QTc interval changes obtained from log-linear corrections optimized for each subject separately. **a**, **c**, and **e** show the experiments of the first type (constant plasma concentrations in a larger group of subjects); **b**, **d**, and **f** show the experiments of the second type (variable plasma concentrations in a smaller group of subjects). In each panel, the distribution of the results in the repeated experiment is shown (see text for details): the red lines show the median value and the pink, green, and blue bands show the ranges between 20th and 80th percentiles, 10th and 90th percentiles, and 5th and 95th percentiles, respectively. The highlighted horizontal line in **c**–**f** shows the standard regulatory threshold of + 10 ms QTc interval change

**Fig. 6:**
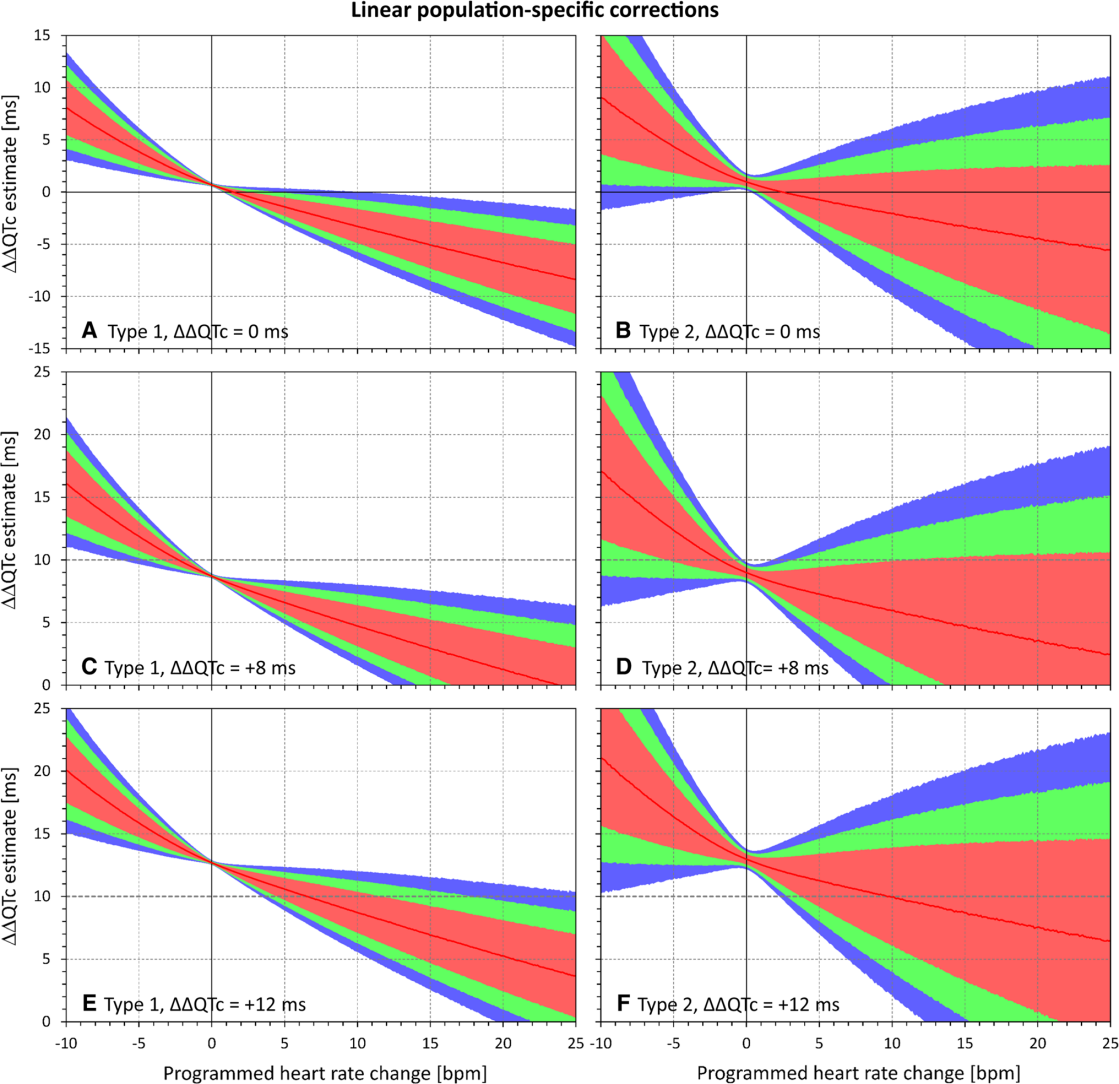
Dependency of the errors of investigated heart rate corrections on the programmed heart rate change. In the experiments shown, heart rate changes ranging from − 10 to + 25 beats per minute (bpm) were combined with programmed corrected QT (QTc) interval changes of 0 ms (**a**, **b**), + 8 ms (**c**, **d**), and + 12 ms (**e**, **f**). The graphs show the distribution of reported QTc interval changes obtained from linear population corrections (optimized for the groups of subjects pooled together). **a**, **c**, and **e** show the experiments of the first type (constant plasma concentrations in a larger group of subjects); **b**, **d**, and **f** show the experiments of the second type (variable plasma concentrations in a smaller group of subjects). In each panel, the distribution of the results in the repeated experiment is shown (see text for details): the red lines show the median value and the pink, green, and blue bands show the ranges between 20th and 80th percentiles, 10th and 90th percentiles, and 5th and 95th percentiles, respectively. The highlighted horizontal line in **c**–**f** shows the standard regulatory threshold of + 10 ms QTc interval change. Compared with Fig. 4, note the substantial increase in the spread of the results

**Fig. 7:**
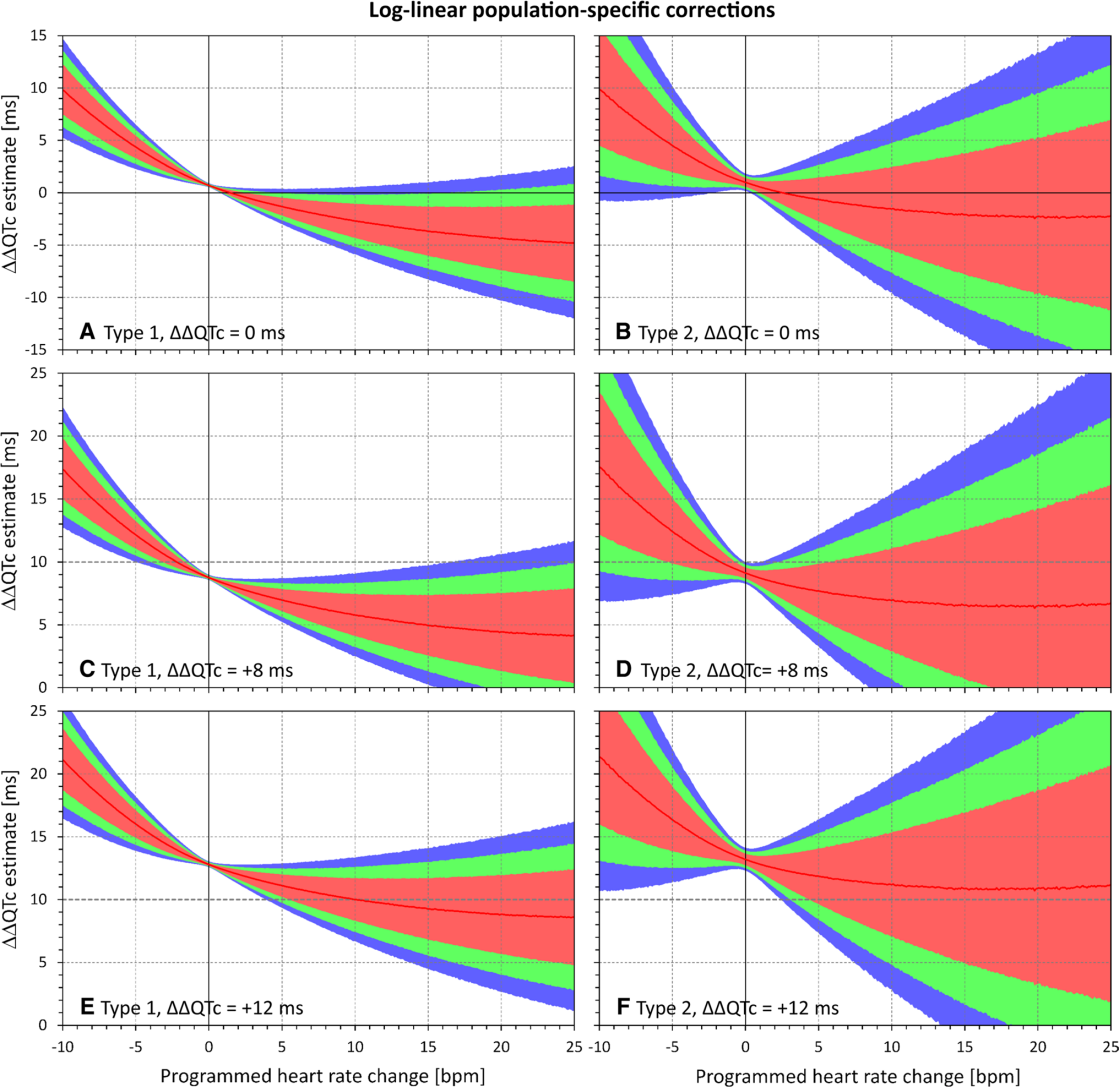
Dependency of the errors of investigated heart rate corrections on the programmed heart rate change. In the experiments shown, heart rate changes ranging from − 10 to + 25 beats per minute (bpm) were combined with programmed corrected QT (QTc) interval changes of 0 ms (**a**, **b**), + 8 ms (**c**, **d**), and + 12 ms (**e**, **f**). The graphs show the distribution of reported QTc interval changes obtained from the log-linear population corrections (optimized for the groups of subjects pooled together). **a**, **c**, and **e** show the experiments of the first type (constant plasma concentrations in a larger group of subjects); **b**, **d**, and **f** show the experiments of the second type (variable plasma concentrations in a smaller group of subjects). In each panel, the distribution of the results in the repeated experiment is shown (see text for details): the red lines show the median value and the pink, green, and blue bands show the ranges between 20th and 80th percentiles, 10th and 90th percentiles, and 5th and 95th percentiles, respectively. The highlighted horizontal line in **c**–**f** shows the standard regulatory threshold of + 10 ms QTc interval change. Compared with Fig. 5, note the substantial increase in the spread of the results

This was confirmed through the spectrum of $\varPhi_{\text{HR}}$ and $\varPhi_{\text{QTc}}$ combinations. Examples of the dependency of ΔΔQTc estimates reported by the different corrections are shown in Figs. 4, 5, 6, and 7. These figures show examples of experiments in which the $\varPhi_{\text{QTc}}$ values were programmed to 0 ms (top row of each these figures), + 8 ms (middle row), and + 12 ms (bottom row), while $\varPhi_{\text{HR}}$ values were considered throughout the whole range between − 10 and 25 bpm. Figures 4, 5, 6, and 7 show the results of differently optimized corrections: linear corrections optimized for each subject separately are shown in Fig. 4; log-linear corrections optimized for each subject separately are shown in Fig. 5; linear corrections optimized for the study population are shown in Fig. 6; and log-linear corrections optimized for the study population are shown in Fig. 7.

The figures confirm the observation made with Figs. 2 and 3. Moreover, the figures also show that the spread of possible ΔΔQTc estimates was much larger with population-specific corrections than with the subject-specific corrections. Not surprisingly, the spread of possible ΔΔQTc estimates was also substantially larger in the experiments of the second type than in the experiments of the first type.

The spread of the results is naturally more important than the median results of all the experiments that cannot be expected to be reached in individual studies. For instance, Figs. 4 and 5 show that if a small clinical study is used to investigate a new drug that causes heart rate increase of 20 bpm, application of subject-specific corrections derived from restricted baselines will lead to ΔΔQTc estimates that can differ by more than 10 ms between different sets of ten subjects. With population corrections (Figs. 6 and 7), the results are completely unpredictable and can differ between different sets of investigated subjects by much more than 25 ms. Note also the dashed horizontal lines in panels c–f of Figs. 4, 5, 6, and 7, which mark the + 10 ms limit of standard regulatory acceptance of ΔΔQTc changes. With population-specific corrections as well as with the individual-specific corrections applied to the experiments of the second type, the results of repeated experiments with the same settings of programmed heart rate and QTc interval changes frequently fall both below and above this limit.

As expected, application of individualized corrections based on full baseline QT/RR data, i.e., corrections in the form ${\text{QTcI}} = {\text{QT}} + (\delta /\gamma )(1 - {\text{RR}}^{\gamma } )$ led to ΔΔQTc estimates that were independent of the $\varPhi_{\text{HR}}$ values [see Electronic Supplementary Material (ESM) 1]. For $\varPhi_{\text{QTc}}$ of 0, + 8, and + 12 ms used in experiments of the first type, these ΔΔQTc estimates were 0.65 ms (90% CI 0.49–0.85), 8.65 ms (90% CI 8.49–8.85), and 12.65 ms (90% CI 12.49–12.85), respectively. In experiments of the second type, the corresponding ΔΔQTc values were 0.92 ms (90% CI 0.15–1.69), 8.92 ms (90% CI 8.15–9.69), and 12.92 ms (90% CI 12.15–13.69).

### Supplementary Analyses

The comparison of linear and log-linear QT/RR slopes obtained in individual subjects from full QT/RR profiles and from the QT/RR data restricted to the selected baseline timepoints are shown in ESM 2. The differences between the full and restricted slopes were statistically significant for both the linear (0.159 ± 0.029 vs. 0.127 ± 0.037) and log-linear (0.344 ± 0.048 vs. 0.306 ± 0.078) slopes (*p* < 0.00001 for both). Compared to the full QT/RR data, the linear and log-linear slopes derived from the selected baseline timepoints were shallower by approximately 20% and 11%, respectively.

The population-specific slopes were also shallower than the averages of the subject-specific slopes (when all were derived from the selected baseline timepoints), as shown in ESM 3. In the 100,000 randomly selected groups of ten subjects, the population-derived linear and log-linear slopes were shallower than the averages of the population slopes by 8.2% and 6.7%, respectively. For the groups of 50 subjects, the corresponding numbers were 7.9% and 6.3%, respectively. Moreover, as seen in ESM 3, the difference between the population slopes and the averages of the individual slopes were also noticeably inconsistent and widely distributed. Examples of populations of ten subjects in which the population-specific slope was substantially different from the average of the subject-specific slopes are shown in ESM 4.

## Discussion

The results of this statistical modeling study offer two principal observations related to the systematic inconsistency and to the inaccuracy of heart rate corrections derived from limited drug-free QT/RR data in individual study subjects.

Although the character of the selected baseline datapoints corresponded well to the data usually obtained in healthy subjects repeatedly placed in supine resting positions [6], the main difference between the selected datapoints and the full QT/RR profiles was in the spread of heart rates covered within each subject. As already mentioned, this was mainly caused by the lack of QT/RR readings at increased heart rates when the data were restricted to the timepoints during which the subjects were per protocol in supine positions. When combined with the inherent variability of imprecision of QT interval measurements, the narrower spread of RR interval values leads to less steep slopes of linear regressions (with narrower ranges of independent variable, of the same level of imprecision of dependent variable has greater shallowing effects on the regression slope). Therefore, compared to the true QT/RR relationship, subject-specific corrections based on restricted baseline data expect the drug-free QT intervals to be longer at faster heart rates and shorter at slower heart rates. Consequently, combined with drug-induced heart rate changes, these corrections are inconsistent, as seen in Fig. 4. The log-linear corrections are additionally complicated by their non-additive properties and by expecting that the true QT/RR relationships are curved differently than the reality. This produced the combined inconsistency seen in Fig. 5, especially the overestimation of the ΔΔQTc estimates at programmed slowing of heart rates and increased variability of the results in experiments of the second type.

The population-specific heart rate corrections suffer from the same problem and, additionally, from the fact that drug-free QT/RR profiles are different in different subjects [2, 12]. Since regression slopes of combined datasets are far from equal to the average of slopes of individual sets [4], the inclination of the population-based slope substantially depends on the individual QT/RR patterns that are combined. While the slopes generally tend to be shallower than the average of individual slopes (because the combination of different individual patterns produces the same effects as regression noise), substantially steeper slopes are also possible. This leads to the increased variability and thus amplified uncertainty of the ΔΔQTc estimates, as seen in Figs. 6 and 7.

Not surprisingly, we observed the variability of ΔΔQTc estimates to be substantially larger in the experiments of the second type, which included only ten subjects each and in which the programmed QT changes followed a changing plasma profile. The difference in the population size was the main reason for the larger spread of possible results of the experiments of the second type than the experiment of the first type which modeled a five times larger population.

To reflect the common practice of population corrections, we used QT/RR regressions of pooled baseline data of all study subjects. Theoretically, a mixed-effect model can also be designed in which the fixed effect represents the group correction, while the corrections applied to individual data involve the sum of fixed and random effects for each subject. This is not being used in practice, probably because if sufficient baseline data exist to allow this type of modeling, they also allow the design of direct individual corrections without any fixed population effect.

The observation that even the accurate individual corrections based on full baseline QT/RR profiles were slightly overestimating the programmed $\varPhi_{\text{QTc}}$ changes of the QTc intervals was caused by the introduction of the $\varepsilon_{\text{QTc}}$ imprecisions. This corresponds to the fact that the standard calculations of the upper CI of ΔΔQTc estimates slightly overestimate the true drug effects [13].

The relatively narrow spreads of results based on linear and log-linear individual-specific corrections (see left-side panels in Figs. 4 and 5) was substantially contributed by the close fit between the modeled RR and QT values. If discrepancies between the RR and QT values exist, e.g., because of the QT/RR hysteresis, the spread of possible results is much larger and includes a substantial proportion of both false positive and false negative findings.

To allow these statistical modeling experiments, we used a published curvilinear description of the drug-free QT/RR relationship in each subject [7]. As previously discussed [2], the use of the drug-free QT/RR relationship to derive an individual-specific heart rate correction formula is based on the knowledge that this relationship is stable and reproducible within each individual. This means that the specific mathematical form used to describe the individual curvilinear QT/RR relationships is of little consequence. Any other regression model that would fit the full drug-free data equally well would clearly lead to very similar if not identical results.

Our results also need to be considered independent of the concentration–QTc interval analysis [9, 14]. While concentration–QTc interval analysis is a powerful tool in the assessment of drug-induced QTc interval changes, its successful application is based on the validity of the QTc interval values. If the investigated drug changes heart rate and if QTc interval values are inconsistent, the application of concentration–QTc interval analysis cannot rectify the correction problems. The same applies to the problems due to the omission of hysteresis correction in concentration–QTc interval analysis [15].

### Limitations

The statistical models that we have used involved several simplifications. While the set-up of the experiments (i.e., the combinations of different programmed heart rate changes and programmed QT changes) was intentionally covering a broad spectrum of possibilities, we have not reproduced any particular heart rate and QT changes observed in a specific previously conducted clinical study.

We have not considered the effects of QT/RR hysteresis [6, 16]. Stable elevated heart rates have different effects on the QT interval compared with rapidly changing and unstable elevated heart rates. Nevertheless, if the association between the baseline drug-free QT intervals and the corresponding RR intervals were inaccurate because of QT/RR hysteresis being ignored, the inconsistency of the investigated corrections would be even larger, although the comparison between individual-specific and population-specific corrections would likely be the same. Since the residuals of the QTcI correction curvatures were very small when using the exponential decay model for the QT/RR hysteresis correction, we have not incorporated other hysteresis models that have been reported to increase the precision of the QT/RR regressions further [17]. In the analyzed data, such more complex models would have little room for improving the QT/RR regression fits.

The experiments used ten baseline datapoints, each with five replicates of QT/RR readings. This might have produced denser QT/RR distributions over wider ranges of heart rates than frequently seen in thorough QT studies that use fewer baseline datapoints and fewer reading replicates [6]. This was unlikely to influence the comparison between subject-specific and population-specific corrections. Nevertheless, the observed variability of the results of the sets of experiments might have been reduced. If fewer baseline datapoints and fewer replicates were used, the widths of the CIs shown in Figs. 2, 3, 4, 5, 6, and 7 would have been wider.

For simplicity, we assumed that inaccuracies $\varepsilon_{\text{HR}}$ and $\varepsilon_{\text{QTc}}$ were equally distributed for all subjects and used relatively narrow bands of the inaccuracies. The variability of the ΔΔQTc estimates of different experiments was therefore likely underestimated. We have not differentiated between female and male subjects. While mixed-sex populations are common in larger clinical studies, early clinical investigations modeled by the experiments of the second type frequently study only males. Nevertheless, when repeating the investigations in sex-specific subgroups (results not shown) we obtained very similar results.

In the experiments of the second type, we have used by-time analysis estimating ΔΔQTc at the maximum modeled drug concentration in each subject. We have used this approach rather than the mixed-effect pharmacokinetic/pharmacodynamic (PK/PD) models [9] since such modeling studies would require further uncertainty coefficients to simulate physiologically realistic plasma profiles. Nevertheless, when we performed limited series of experiments with mixed-effects PK/PD models (not shown), the comparisons of the individual-specific and population-specific corrections were practically the same as the results that we presented with the same wide spread and uncertainty of the of ΔΔQTc values provided by the population corrections. This is not surprising since the increased uncertainty is caused by combinations of subjects in whom the population corrections underestimate and overestimate the QT/RR slopes (and thus underestimate and overestimate the QTc interval values expected at increased heart rates) rather than by any differences in QTc interval data processing methods. The experiments of the second type also assume that there is no PK/PD hysteresis, i.e., that the changes of the QT intervals and of heart rate followed the plasma profile without any delay. Situations when such a delay exists and when it is different for the QT interval and for heart rate are possible. However, such situations would again disconnect the QT and heart rate measurements further, thus increasing the inconsistency of the population corrections.

The models were based on data obtained in healthy subjects. We cannot directly comment on the implication of the results for studies involving diseased populations. Nevertheless, since diseased patients are unlikely to have lesser variability of QT intervals at the same heart rates, the variability and inconsistency of the investigated corrections would likely be even greater in such studies.

Finally, our experiments considered combinations of programmed heart rate and QT interval changes. We have not considered secondary QT interval prolongation due to drug-induced QRS widening, such as by sodium channel antagonists and other drugs [18, 19]. For the same reason, our results are not directly applicable to studies in patients with intra-ventricular conduction abnormalities.

## Conclusions

Despite these limitations, the study shows that subject-specific heart rate corrections based on limited baseline drug-free data may lead to inconsistent results and, in the presence of underlying heart rate changes, may potentially underestimate or overestimate ΔΔQTc changes. This confirms the criticism of these corrections made in the guidance by the Cardiac Safety Research Consortium [2].

The population-specific heart rate corrections are even more problematic. As seen in Figs. 6 and 7, they not only show (on average) similar potential inconsistency as the subject-specific corrections, but their ΔΔQTc estimates were also widely distributed in repeated experiments. This is of particular concern in small clinical studies. For instance, Fig. 6f shows that when true QTc interval increase of 12 ms was combined with heart rate changes of 20 bpm, not only did a substantial proportion (well above 50%!) of the experiments yield an estimated ΔΔQTc below the regulatory threshold of 10 ms but almost 10% of the experiments resulted in ΔΔQTc below 0. When the 90% CI of the repeated ΔΔQTc estimates exceeds the range of 0–25 ms (see Fig. 7d, f), the method is of no practical value.

Because of the substantial variability of possible results, the study also suggests that the disadvantages of the population-specific corrections are greater than those of the subject-specific corrections. Nevertheless, this cannot be interpreted as endorsement of subject-specific corrections based on limited baseline data (in real studies, the imprecision of QT and RR values is larger than in these models). Similarly, irrespective of whether the investigated drugs change heart rate or not, clinical studies do not result in stable heart rate levels [6]. While we have not considered variable heart rate changes in these experiments, it is always important to account for QT/RR hysteresis [6].

For accurate ΔΔQTc estimates in the presence of substantial drug-induced heart rate changes, subject-specific corrections based on drug-free QT/RR profiles involving wide heart rate spans are needed [2]. This is particularly true for clinical studies involving fewer subjects (such as those modeled by our experiments of the second type). It is also known that other previously suggested approaches to estimate QTc interval changes combined with heart rate changes, such as the so-called bin method [20], are in principle equivalent to the full heart rate range QT/RR profiles [21].

## Electronic supplementary material

Below is the link to the electronic supplementary material.

Supplementary material 1 (PDF 829 kb)

Supplementary material 2 (PDF 771 kb)

Supplementary material 3 (PDF 1991 kb)

Supplementary material 4 (PDF 1637 kb)
