# Correction to: Implications of Individual QT/RR Profiles—Part 1: Inaccuracies and Problems of Population-Specific QT/Heart Rate Corrections

**DOI:** 10.1007/s40264-019-00801-w

**Published:** 2019-03-09

**Authors:** Marek Malik, Christine Garnett, Katerina Hnatkova, Jose Vicente, Lars Johannesen, Norman Stockbridge

**Affiliations:** 1 National Heart and Lung Institute, Imperial College, Dovehouse Street, London, SW3 6LY England, UK; 2 Division of Cardiovascular and Renal Products, Office of New Drugs, Center for Drug Evaluation and Research, US Food and Drug Administration, Silver Spring, MD USA; 3 Division of Clinical Pharmacology I, Office of Clinical Pharmacology, Center for Drug Evaluation and Research, US Food and Drug Administration, Silver Spring, MD USA

## Correction to: Drug Safety 10.1007/s40264-018-0736-1

The Open Access license, which previously read:

**Open Access** This article is distributed under the terms of the Creative Commons Attribution-NonCommercial 4.0 International License (http://creativecommons.org/licenses/by-nc/4.0/), which permits any noncommercial use, distribution, and reproduction in any medium, provided you give appropriate credit to the original author(s) and the source, provide a link to the Creative Commons license, and indicate if changes were made.

Should read:

**Open Access** This article is distributed under the terms of the Creative Commons Attribution 4.0 International License (http://creativecommons.org/licenses/by/4.0/), which permits unrestricted use, distribution, and reproduction in any medium, provided you give appropriate credit to the original author(s) and the source, provide a link to the Creative Commons license, and indicate if changes were made.

