# Supplementary material for: Implications of Individual QT/RR Profiles—Part 1: Inaccuracies and Problems of Population-Specific QT/Heart Rate Corrections
Source: Drug Saf. 2018 Sep 25;42(3):401–14. doi: 10.1007/s40264-018-0736-1 (PMC6426828; doi:10.1007/s40264-018-0736-1)
Supplement: Supplementary file 1 — Supplementary material 1 (PDF 829 kb) [file 40264_2018_736_MOESM1_ESM.pdf]

Article title: Implications of individual QT/RR profiles
Part 1: Inaccuracies and problems of population-specific QT/heart rate corrections
Journal name: Drug Safety
Author names: Marek Malik (corresponding), Christine Garnett, Katerina Hnatkova, Jose Vicente, Lars Johannesen, Norman Stockbridge
Affiliation of corresponding author: National Heart and Lung Institute, Imperial College, Dovehouse Street, London SW3 6LY, England
Email of corresponding author: marek.malik@btinternet.com / marek.malik@imperial.ac.uk

Electronic Supplementary Material 1

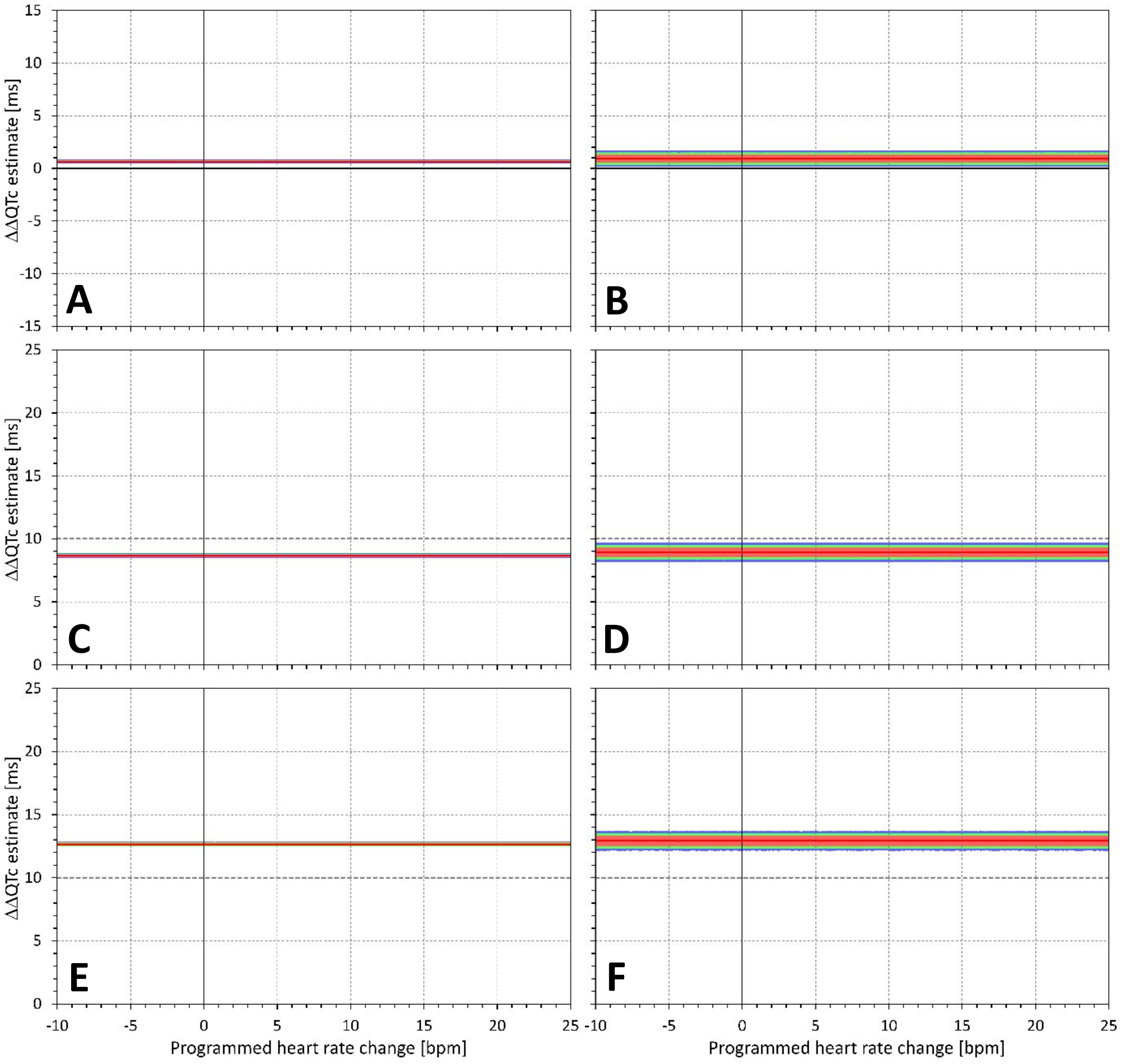

The layout of the figure is the same as of Figures 4 to 7 in the main manuscript. The Figure shows the individual-specific corrections in the form $QTcI = QT + (\delta/\gamma)(1 - RR^{\gamma})$ optimized on the full baseline QT/RR data.
