# Supplementary material for: Implications of Individual QT/RR Profiles—Part 1: Inaccuracies and Problems of Population-Specific QT/Heart Rate Corrections
Source: Drug Saf. 2018 Sep 25;42(3):401–14. doi: 10.1007/s40264-018-0736-1 (PMC6426828; doi:10.1007/s40264-018-0736-1)
Supplement: Supplementary file 2 — Supplementary material 2 (PDF 771 kb) [file 40264_2018_736_MOESM2_ESM.pdf]

Article title: Implications of individual QT/RR profiles
Part 1: Inaccuracies and problems of population-specific QT/heart rate corrections
Journal name: Drug Safety
Author names: Marek Malik (corresponding), Christine Garnett, Katerina Hnatkova, Jose Vicente, Lars Johannesen, Norman Stockbridge
Affiliation of corresponding author: National Heart and Lung Institute, Imperial College, Dovehouse Street, London SW3 6LY, England
Email of corresponding author: marek.malik@btinternet.com / marek.malik@imperial.ac.uk

Electronic Supplementary Material 2

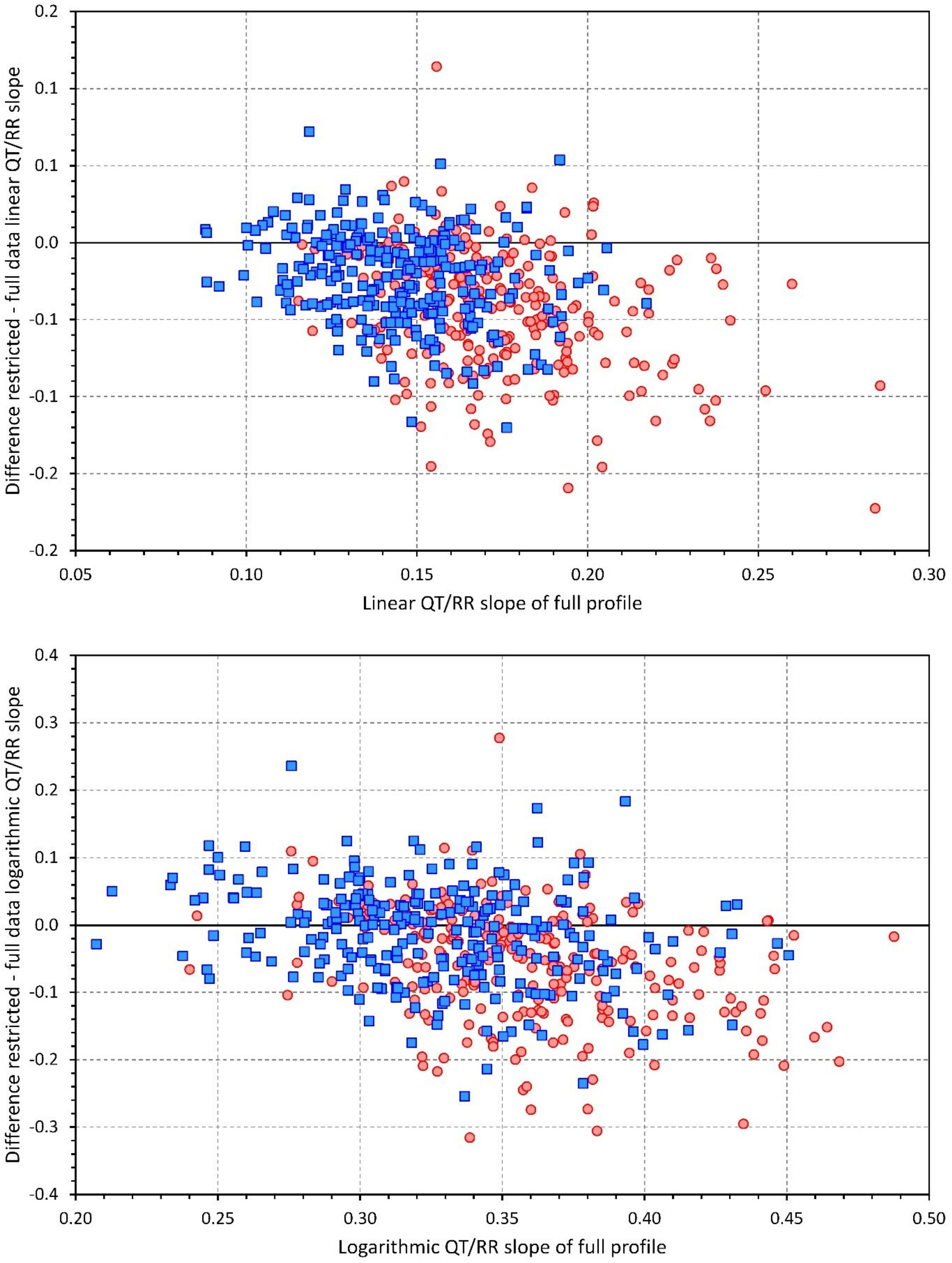

The top panel shows the intra-subject differences between the linear QT/RR slopes assessed from the data restricted to the selected baseline time points and the full profile data (vertical axis) versus the full profile linear QT/RR slopes (horizontal axis). The bottom panel shows the same for the log-linear slopes. In both panels, red circles and blue squares correspond to female and male subjects, respectively.
