# Supplementary material for: Implications of Individual QT/RR Profiles—Part 1: Inaccuracies and Problems of Population-Specific QT/Heart Rate Corrections
Source: Drug Saf. 2018 Sep 25;42(3):401–14. doi: 10.1007/s40264-018-0736-1 (PMC6426828; doi:10.1007/s40264-018-0736-1)
Supplement: Supplementary file 3 — Supplementary material 3 (PDF 1991 kb) [file 40264_2018_736_MOESM3_ESM.pdf]

Article title: Implications of individual QT/RR profiles
Part 1: Inaccuracies and problems of population-specific QT/heart rate corrections
Journal name: Drug Safety
Author names: Marek Malik (corresponding), Christine Garnett, Katerina Hnatkova, Jose Vicente, Lars Johannesen, Norman Stockbridge
Affiliation of corresponding author: National Heart and Lung Institute, Imperial College, Dovehouse Street, London SW3 6LY, England
Email of corresponding author: marek.malik@btinternet.com / marek.malik@imperial.ac.uk

Electronic Supplementary Material 3

Part 1

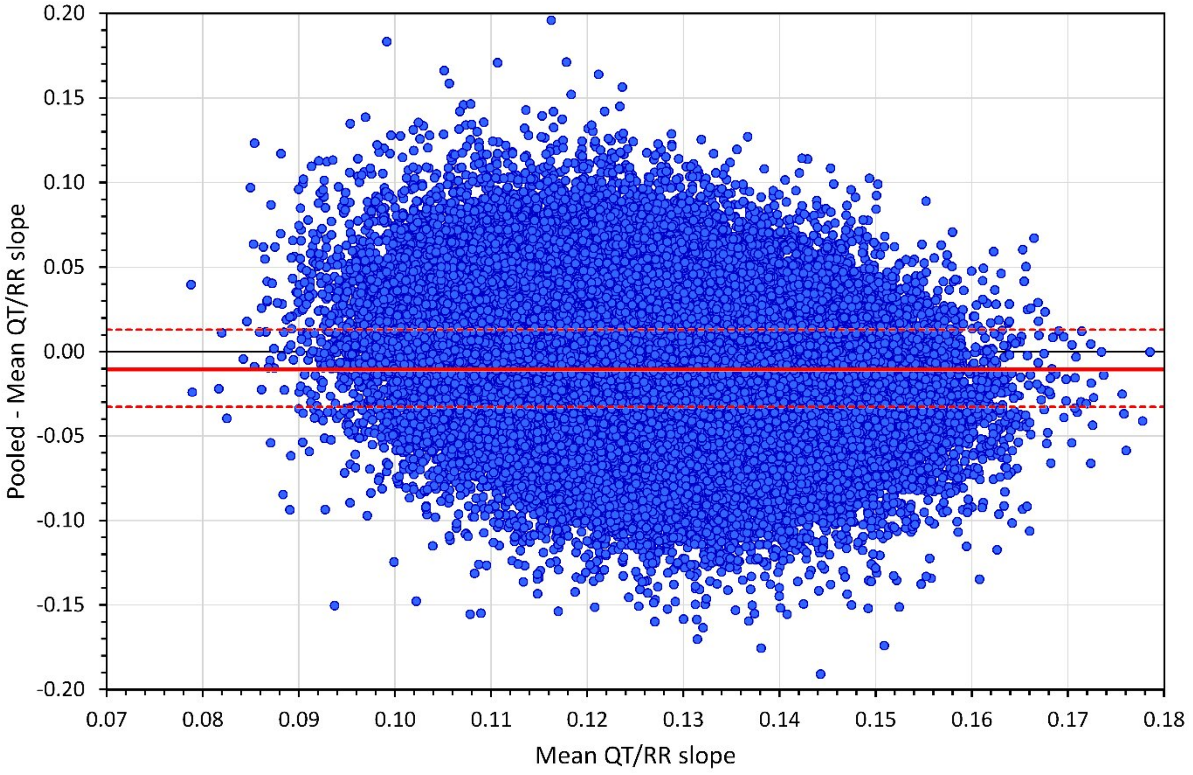

0.20
0.15
0.10
0.05
0.00
-0.05
-0.10
-0.15
-0.20
Pooled - Mean QT/RR slope
0.07
0.08
0.09
0.10
0.11
0.12
0.13
0.14
0.15
0.16
0.17
0.18
Mean QT/RR slope

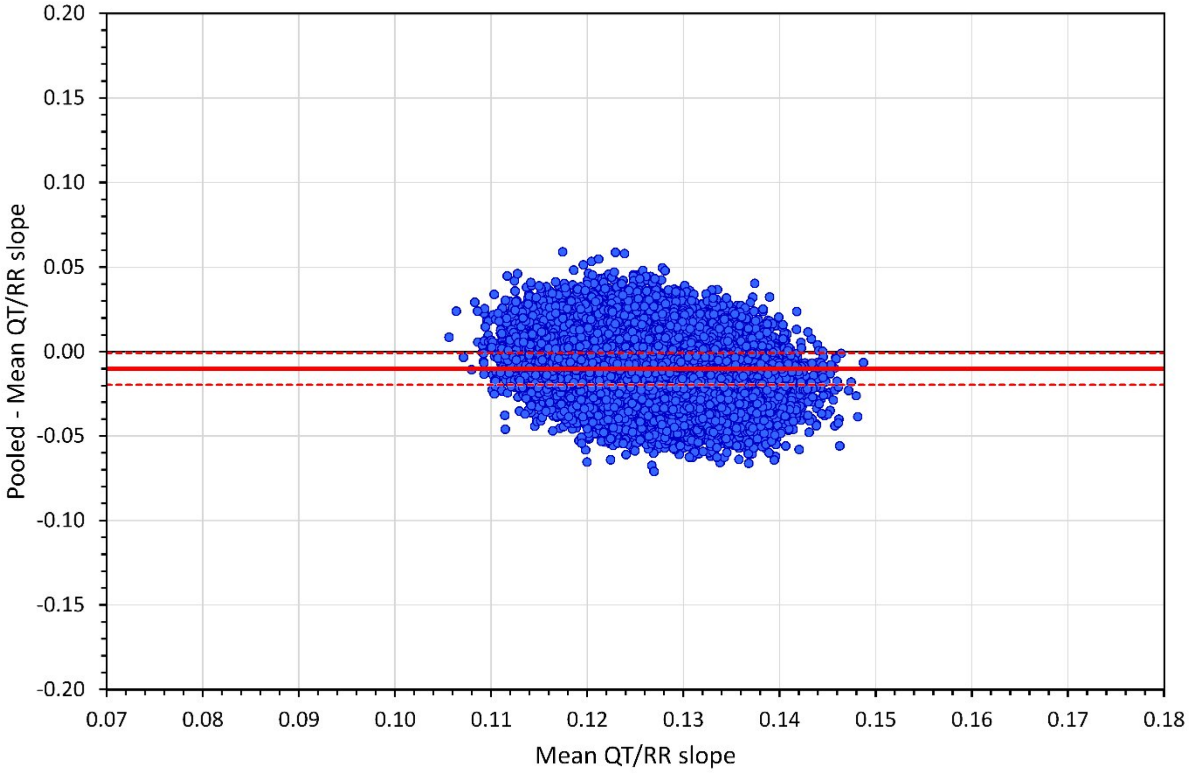

0.20
0.15
0.10
0.05
0.00
-0.05
-0.10
-0.15
-0.20
Pooled - Mean QT/RR slope
0.07
0.08
0.09
0.10
0.11
0.12
0.13
0.14
0.15
0.16
0.17
0.18
Mean QT/RR slope

Part 2

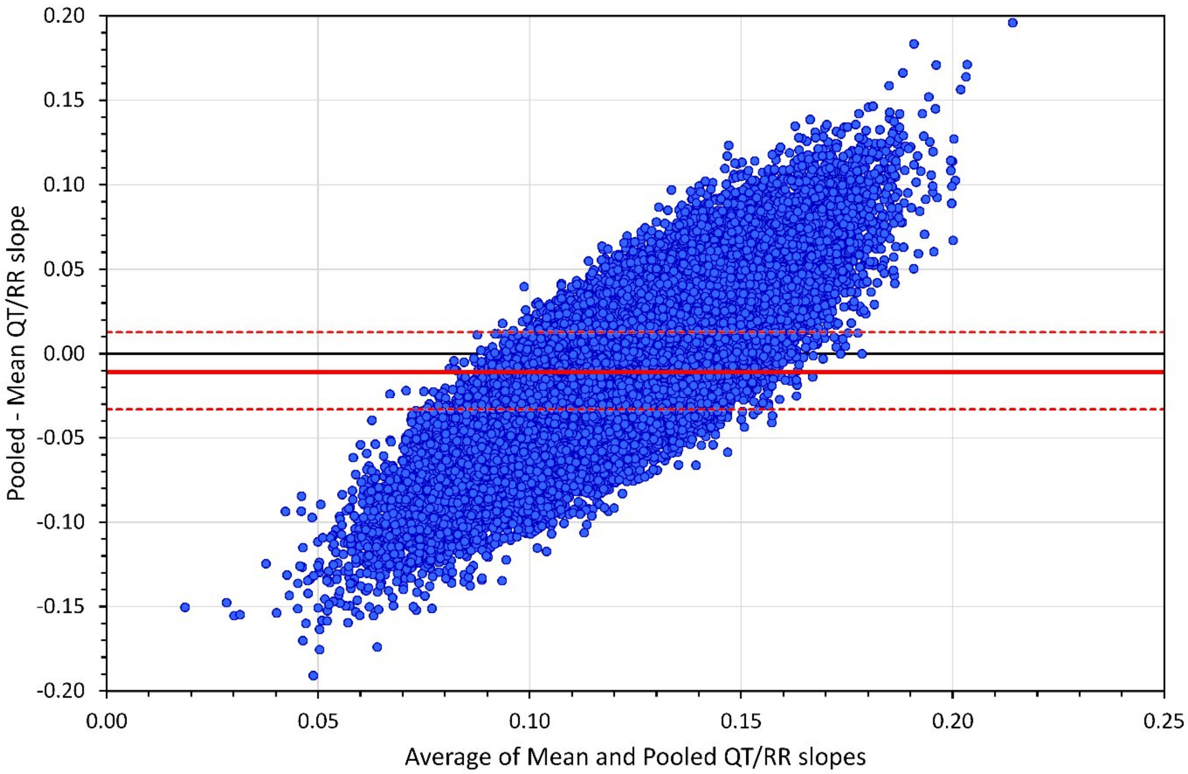
Pooled - Mean QT/RR slope
Average of Mean and Pooled QT/RR slopes
0.20
0.15
0.10
0.05
0.00
-0.05
-0.10
-0.15
-0.20
0.00
0.05
0.10
0.15
0.20
0.25

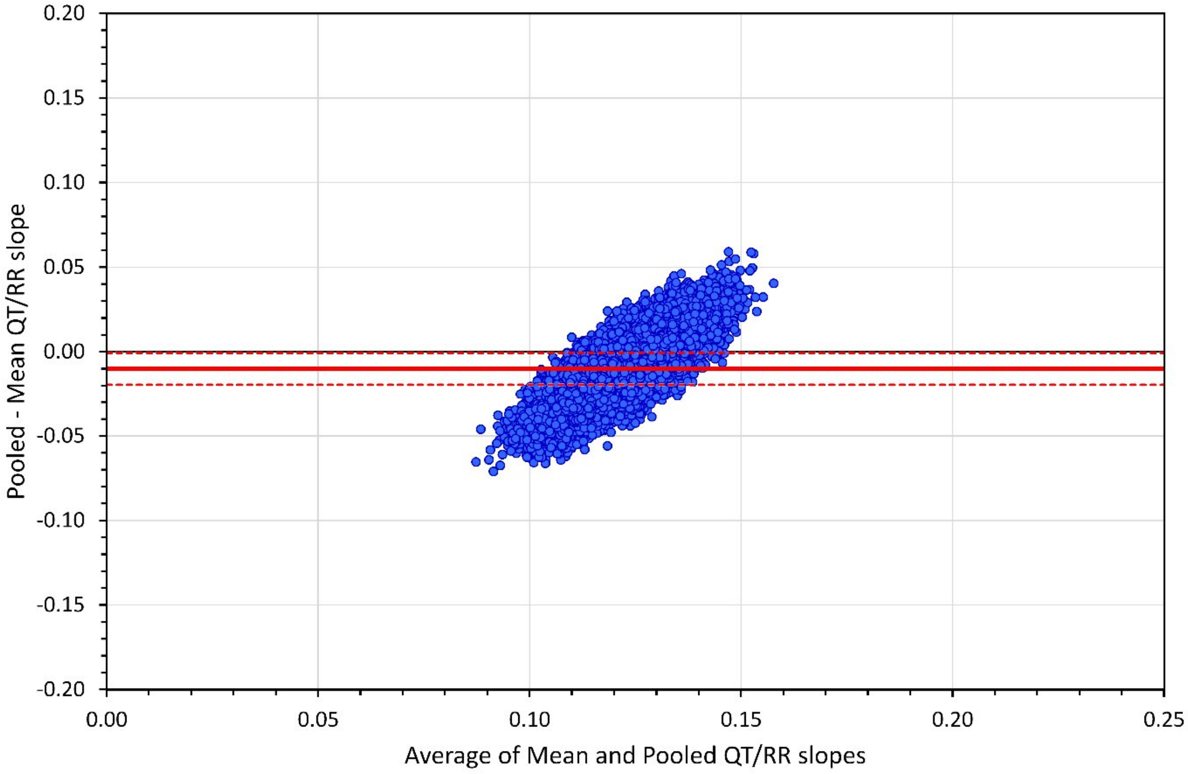
Pooled - Mean QT/RR slope
Average of Mean and Pooled QT/RR slopes
0.20
0.15
0.10
0.05
0.00
-0.05
-0.10
-0.15
-0.20
0.00
0.05
0.10
0.15
0.20
0.25

Part 3

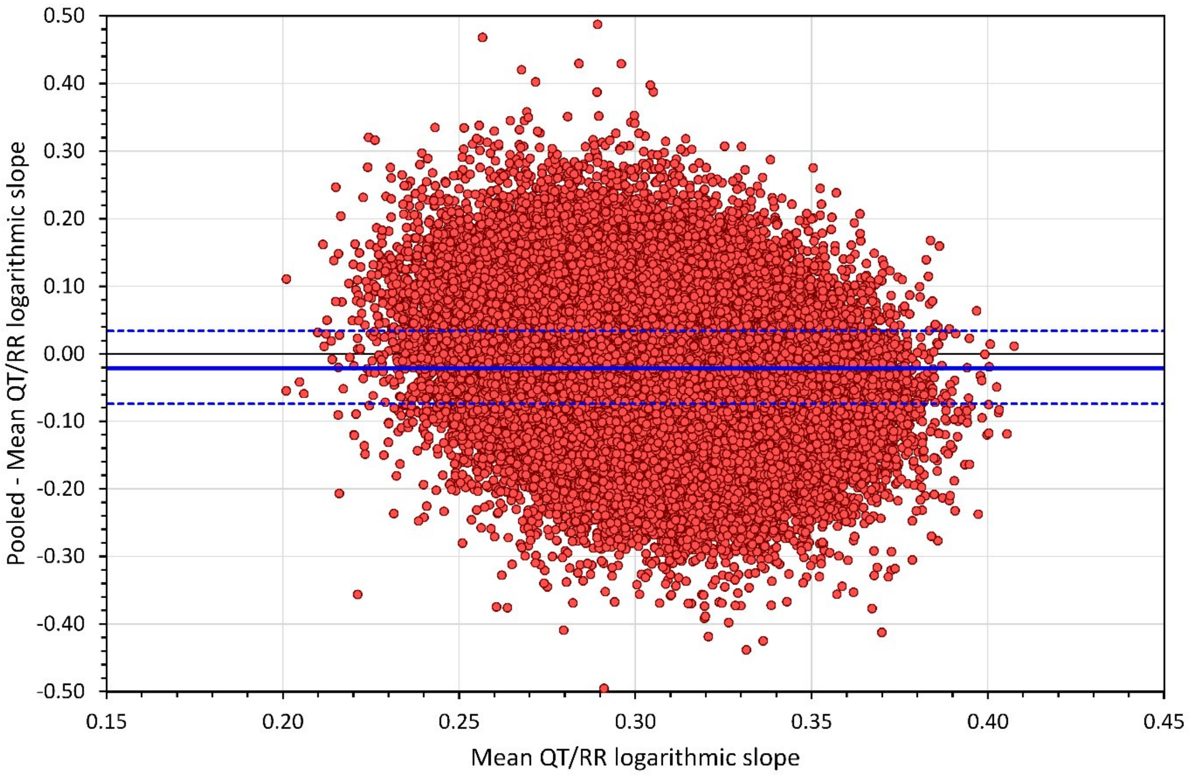

0.50
0.40
0.30
0.20
0.10
0.00
-0.10
-0.20
-0.30
-0.40
-0.50
Pooled - Mean QT/RR logarithmic slope
0.15
0.20
0.25
0.30
0.35
0.40
0.45
Mean QT/RR logarithmic slope

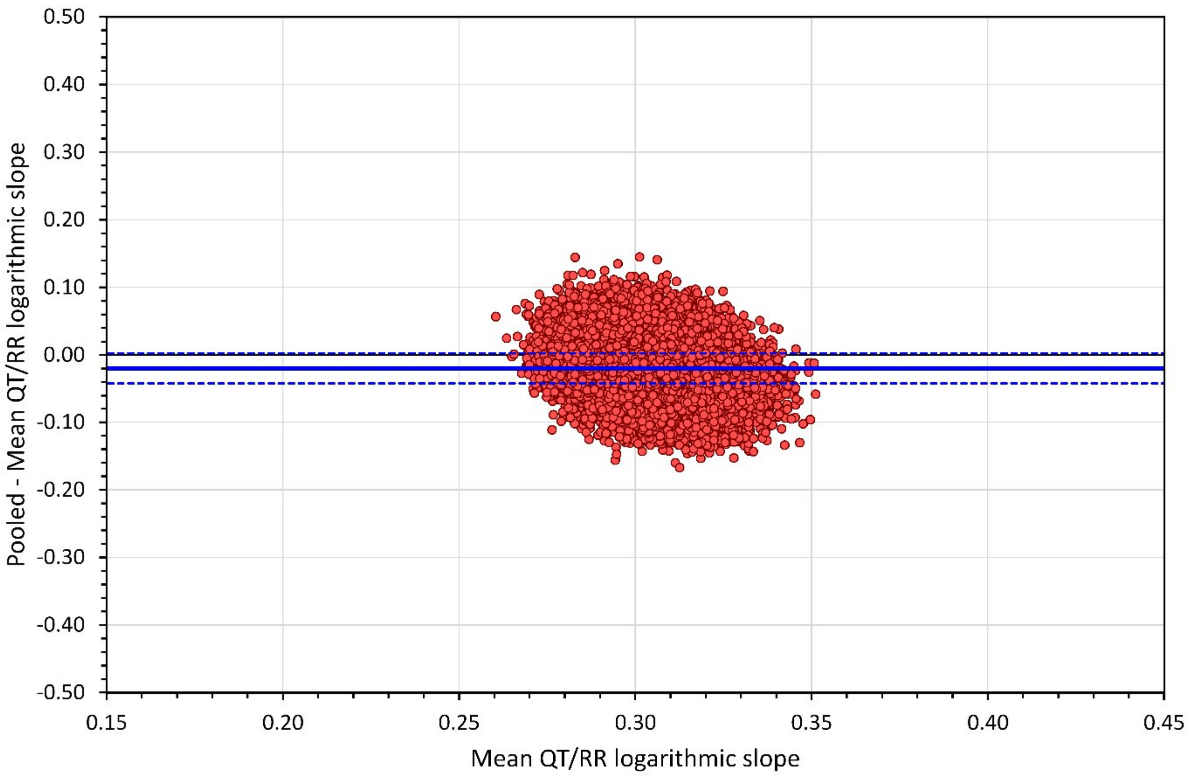

0.50
0.40
0.30
0.20
0.10
0.00
-0.10
-0.20
-0.30
-0.40
-0.50
Pooled - Mean QT/RR logarithmic slope
0.15
0.20
0.25
0.30
0.35
0.40
0.45
Mean QT/RR logarithmic slope

Part 4

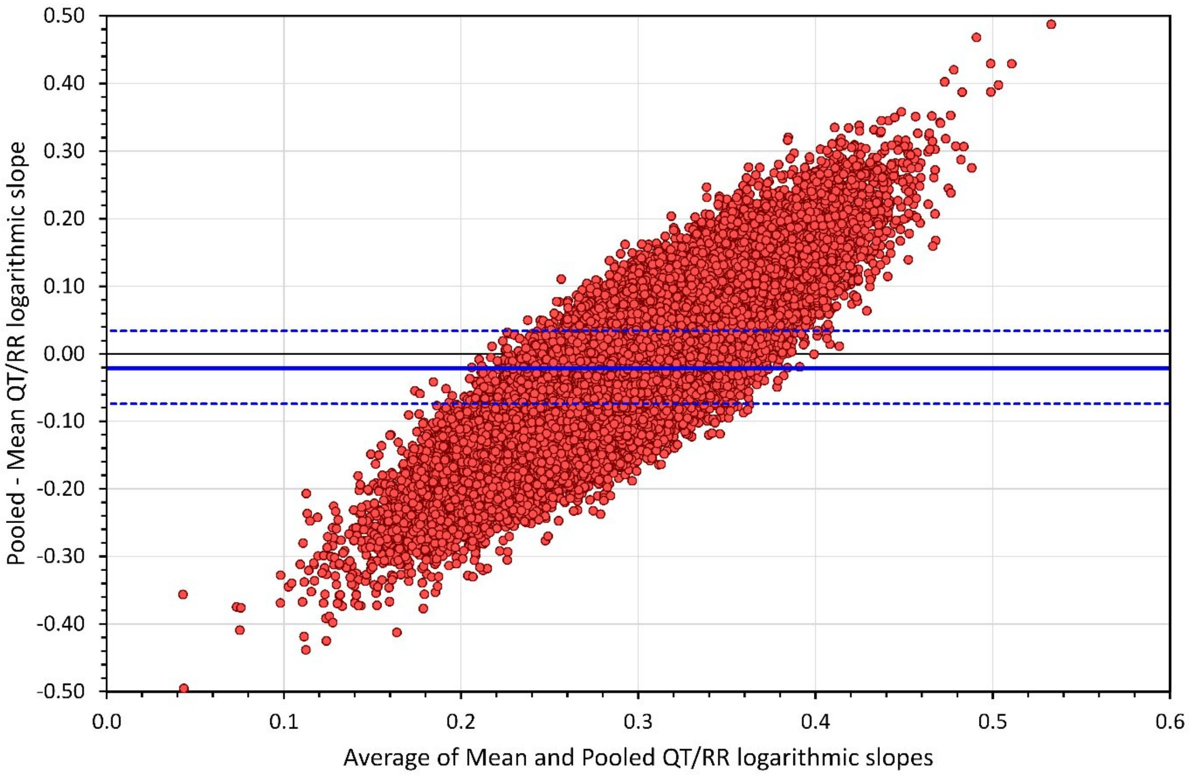

0.50
0.40
0.30
0.20
0.10
0.00
-0.10
-0.20
-0.30
-0.40
-0.50
Pooled - Mean QT/RR logarithmic slope
0.0
0.1
0.2
0.3
0.4
0.5
0.6
Average of Mean and Pooled QT/RR logarithmic slopes

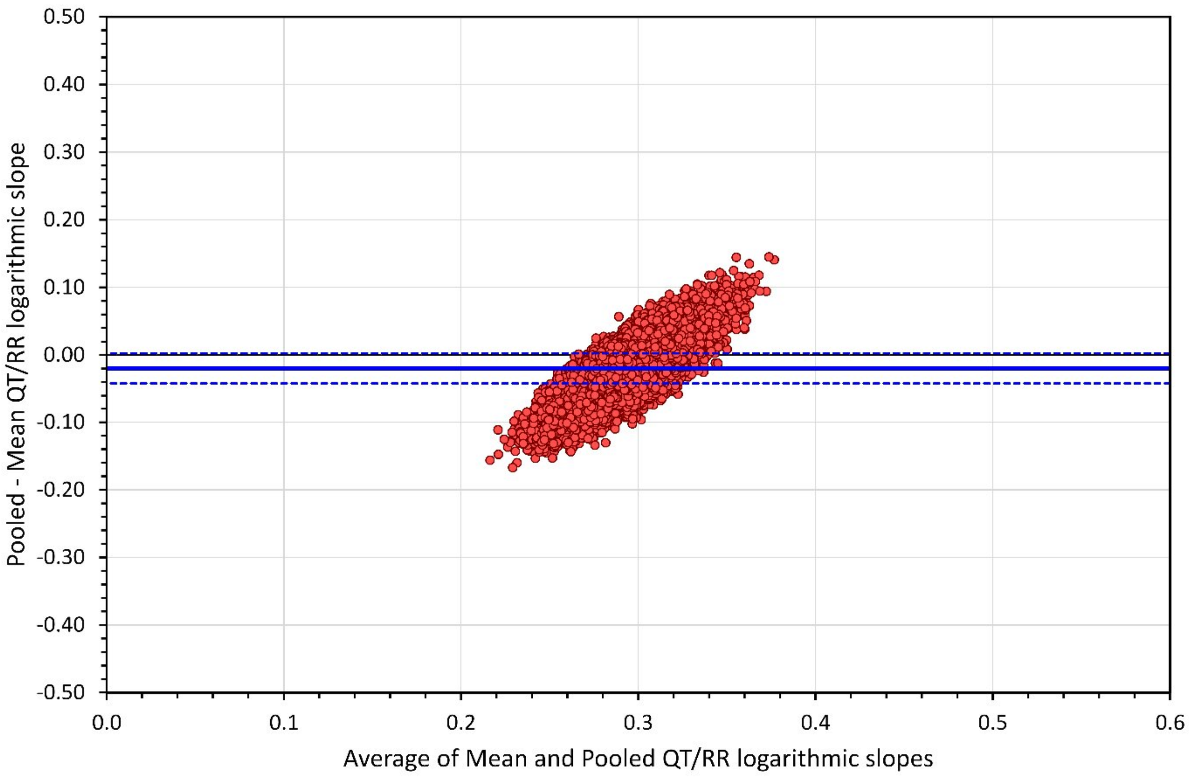

0.50
0.40
0.30
0.20
0.10
0.00
-0.10
-0.20
-0.30
-0.40
-0.50
Pooled - Mean QT/RR logarithmic slope
0.0
0.1
0.2
0.3
0.4
0.5
0.6
Average of Mean and Pooled QT/RR logarithmic slopes

The electronic supplementary material shows the differences between population-specific QT/RR slopes (derived from the selected baseline QT/RR data pooled from several subjects) and the averages of subject-specific QT/RR slopes (derived from the selected QT/RR data of each subject separately). The material consists of 4 parts, each of two panels.

Part 1 shows the differences between the population-specific (pooled) linear QT/RR slope and the averages of individual linear QT/RR slopes (horizontal axis) versus the mean of individual linear QT/RR slopes (horizontal axis). The 100,000 repetitions of the populations of 10 and 50 subjects are shown in the top and bottom panel, respectively.

Part 2 show the differences between the population-specific (pooled) linear QT/RR slope and the averages of individual linear QT/RR slopes (horizontal axis) versus the averaged value of the population-specific linear slope and of the mean of individual linear QT/RR slopes (horizontal axis). The 100,000 repetitions of the populations of 10 and 50 subjects are shown in the top and bottom panel, respectively.

Part 3 shows the differences between the population-specific (pooled) log-linear QT/RR slope and the averages of individual log-linear QT/RR slopes (horizontal axis) versus the mean of individual log-linear QT/RR slopes (horizontal axis). The 100,000 repetitions of the populations of 10 and 50 subjects are shown in the top and bottom panel, respectively.

Part 4 show the differences between the population-specific (pooled) log-linear QT/RR slope and the averages of individual log-linear QT/RR slopes (horizontal axis) versus the averaged value of the population-specific log-linear slope and of the mean of log-individual linear QT/RR slopes (horizontal axis). The 100,000 repetitions of the populations of 10 and 50 subjects are shown in the top and bottom panel, respectively.

In all panels, the full horizontal line shows the median of the differences between the population-specific slopes and the means of the subject-specific slopes. The dashed horizontal lines show the inter-quartile range of the differences.

Note also in Parts 1 and 3 that the differences between the population-specific slopes and the means of the subject-specific slopes are negatively correlated to the means of the subject-specific slopes.
