# Supplementary material for: Implications of Individual QT/RR Profiles—Part 1: Inaccuracies and Problems of Population-Specific QT/Heart Rate Corrections
Source: Drug Saf. 2018 Sep 25;42(3):401–14. doi: 10.1007/s40264-018-0736-1 (PMC6426828; doi:10.1007/s40264-018-0736-1)
Supplement: Supplementary file 4 — Supplementary material 4 (PDF 1637 kb) [file 40264_2018_736_MOESM4_ESM.pdf]

Article title: Implications of individual QT/RR profiles
Part 1: Inaccuracies and problems of population-specific QT/heart rate corrections
Journal name: Drug Safety
Author names: Marek Malik (corresponding), Christine Garnett, Katerina Hnatkova, Jose Vicente, Lars Johannesen, Norman Stockbridge
Affiliation of corresponding author: National Heart and Lung Institute, Imperial College, Dovehouse Street, London SW3 6LY, England
Email of corresponding author: marek.malik@btinternet.com / marek.malik@imperial.ac.uk

Electronic Supplementary Material 4

Part 1

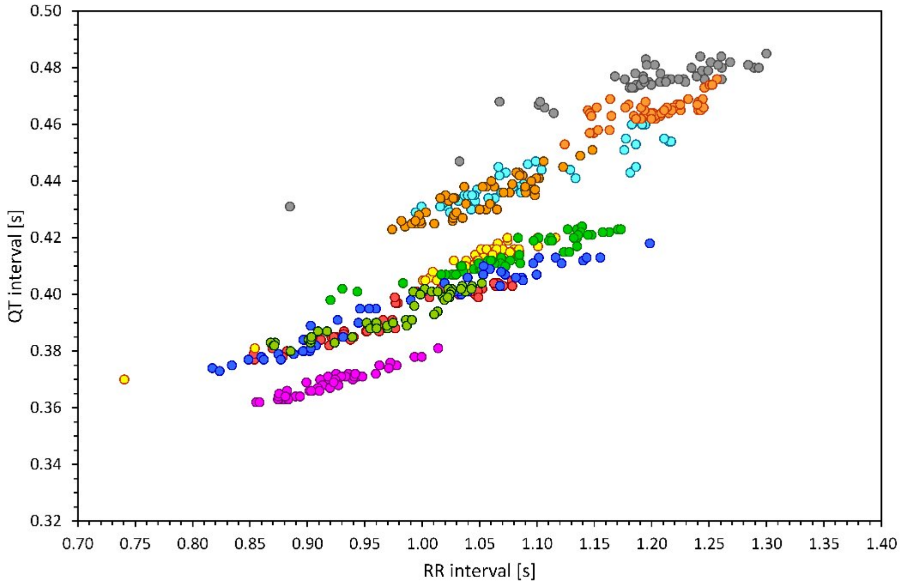

0.50
0.48
0.46
0.44
0.42
0.40
0.38
0.36
0.34
0.32
QT interval [s]
0.70 0.75 0.80 0.85 0.90 0.95 1.00 1.05 1.10 1.15 1.20 1.25 1.30 1.35 1.40
RR interval [s]

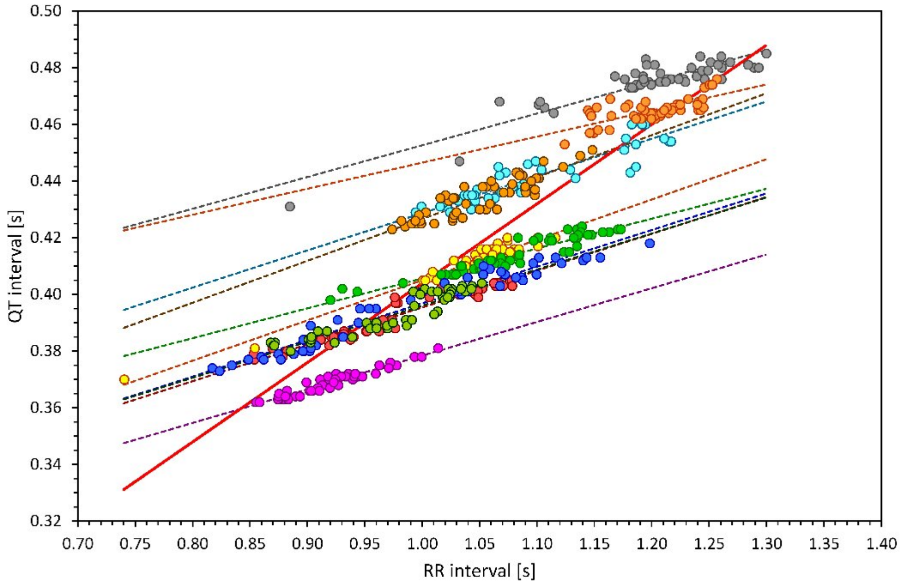

0.50
0.48
0.46
0.44
0.42
0.40
0.38
0.36
0.34
0.32
QT interval [s]
0.70 0.75 0.80 0.85 0.90 0.95 1.00 1.05 1.10 1.15 1.20 1.25 1.30 1.35 1.40
RR interval [s]

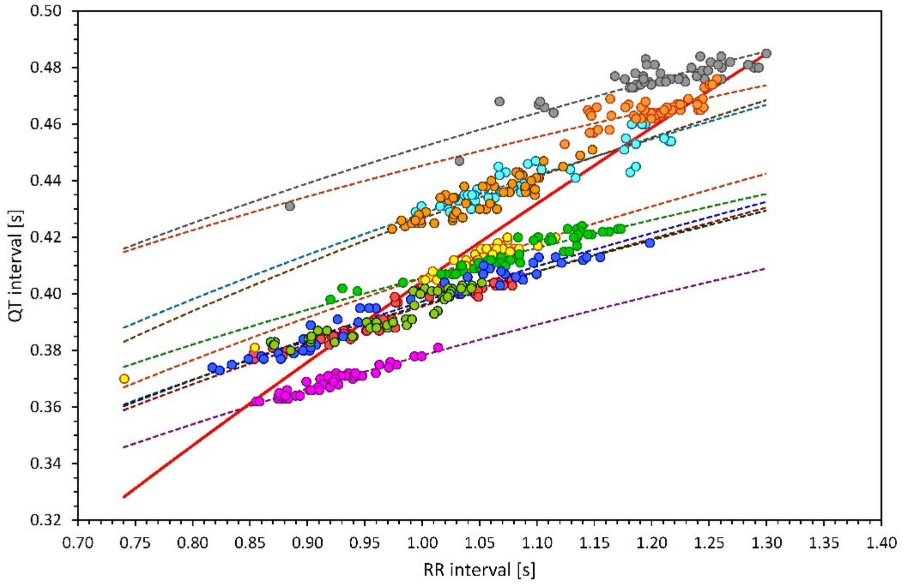

0.50
0.48
0.46
0.44
0.42
0.40
0.38
0.36
0.34
0.32
QT interval [s]
0.70 0.75 0.80 0.85 0.90 0.95 1.00 1.05 1.10 1.15 1.20 1.25 1.30 1.35 1.40
RR interval [s]

Part 2

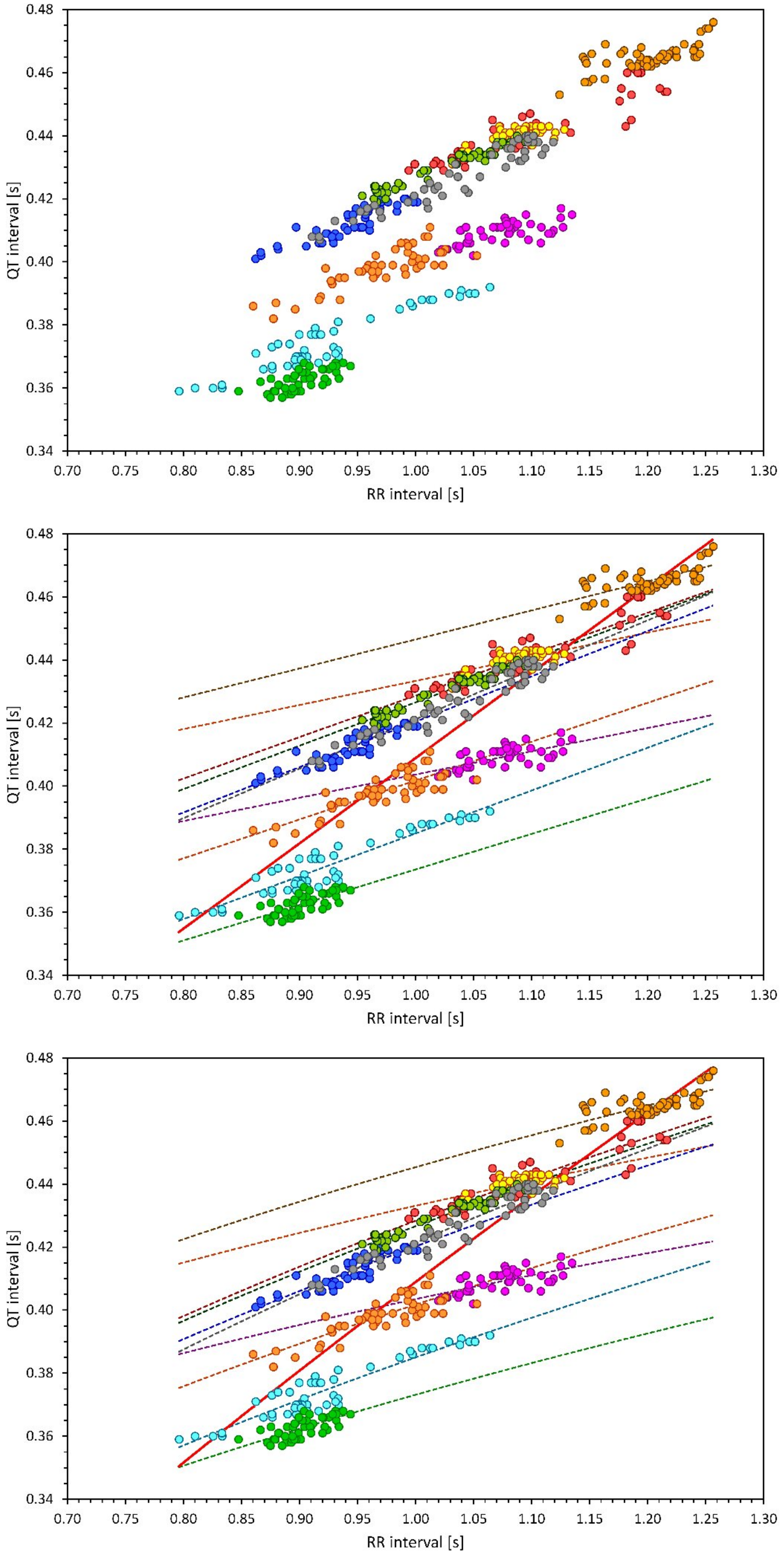
QT interval [s]
RR interval [s]
0.34
0.36
0.38
0.40
0.42
0.44
0.46
0.48
0.70
0.75
0.80
0.85
0.90
0.95
1.00
1.05
1.10
1.15
1.20
1.25
1.30

Part 3

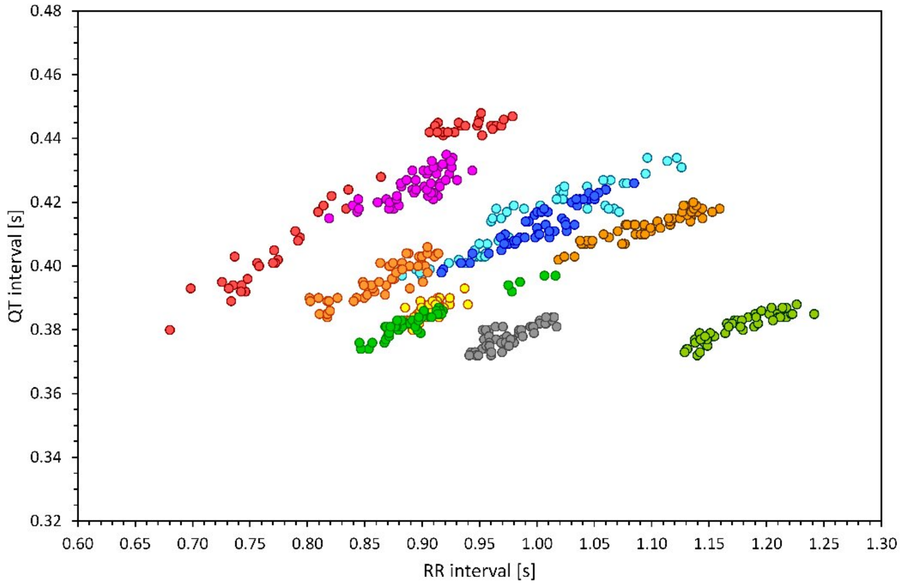
0.48
0.46
0.44
0.42
0.40
0.38
0.36
0.34
0.32
QT interval [s]
0.60 0.65 0.70 0.75 0.80 0.85 0.90 0.95 1.00 1.05 1.10 1.15 1.20 1.25 1.30
RR interval [s]

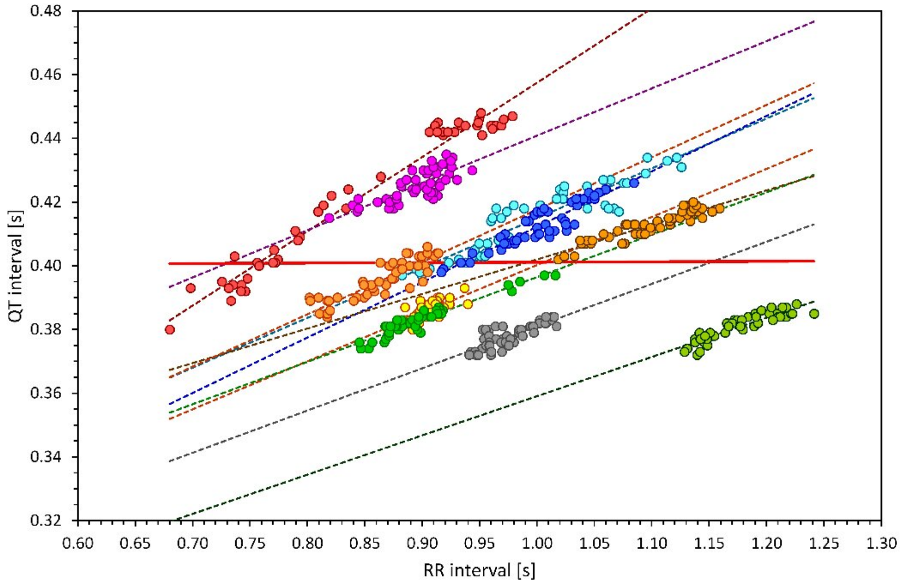
0.48
0.46
0.44
0.42
0.40
0.38
0.36
0.34
0.32
QT interval [s]
0.60 0.65 0.70 0.75 0.80 0.85 0.90 0.95 1.00 1.05 1.10 1.15 1.20 1.25 1.30
RR interval [s]

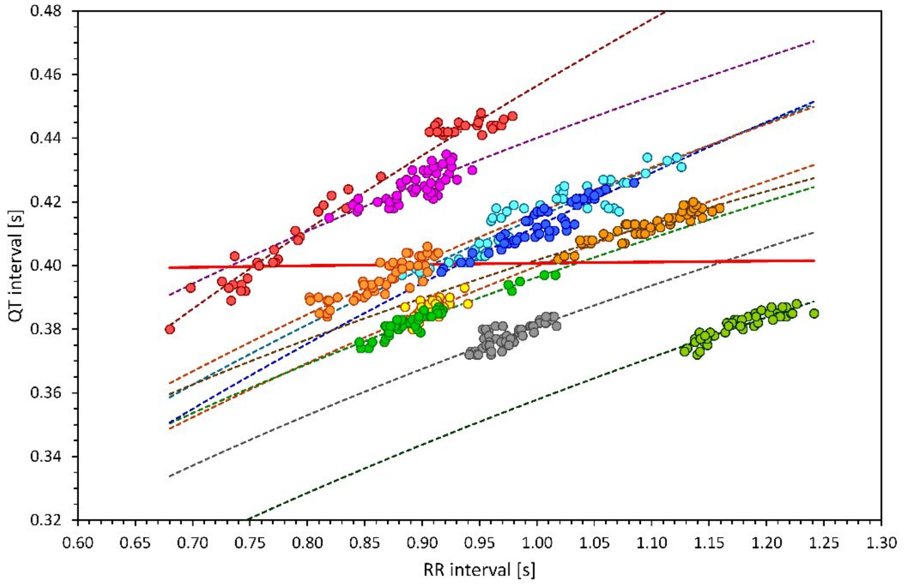
0.48
0.46
0.44
0.42
0.40
0.38
0.36
0.34
0.32
QT interval [s]
0.60 0.65 0.70 0.75 0.80 0.85 0.90 0.95 1.00 1.05 1.10 1.15 1.20 1.25 1.30
RR interval [s]

Part 4

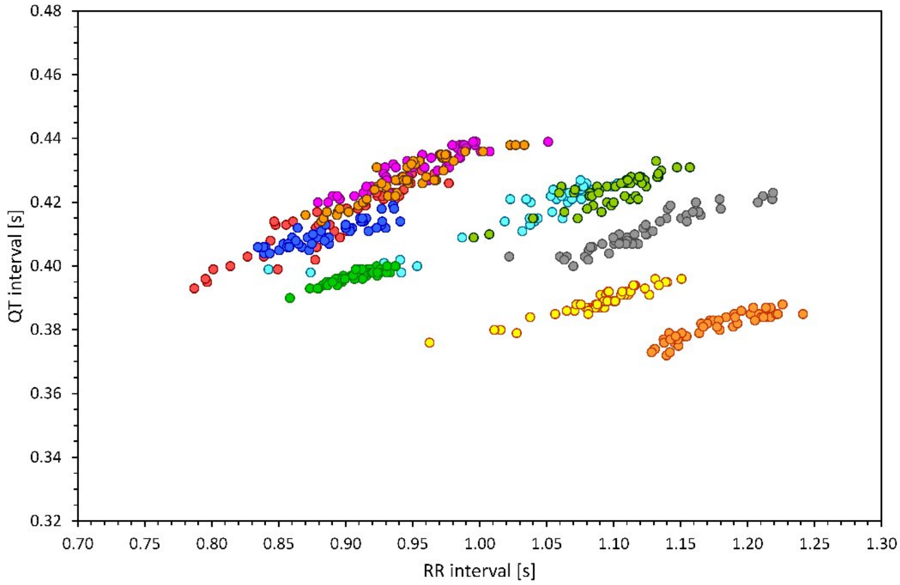

QT interval [s]
RR interval [s]

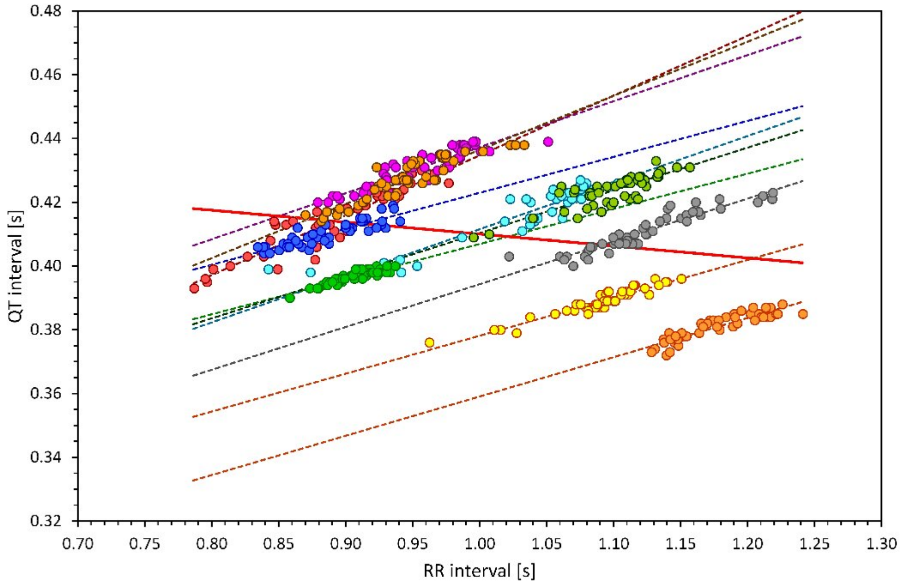

QT interval [s]
RR interval [s]

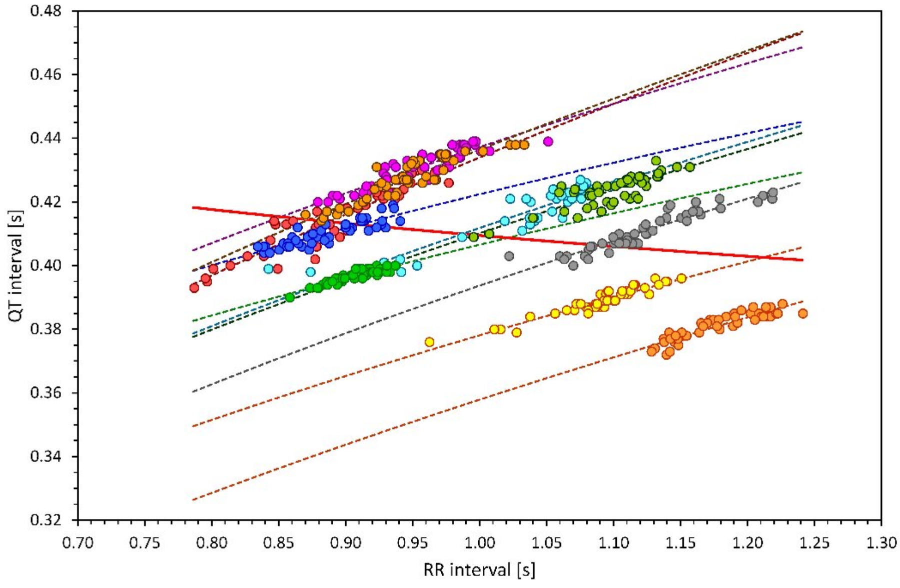

QT interval [s]
RR interval [s]

The electronic supplementary material shows 4 examples (Parts 1 to 4) of substantial differences between subject-specific slopes and population-specific slopes in different populations of 10 subjects. In each part, 3 panels are shown.

The top panel shows the QT/RR data of selected baseline time-points in 10 different subjects (distinguished by different colors). The middle panel shows linear QT/RR regression of the baseline time-points for each subject separately (short dashed lines of the corresponding colors) and of the baseline time-points pooled from all 10 subjects together (bold red line). The bottom panel shows log-linear QT/RR regression of the baseline time-points for each subject separately (short dashed lines of the corresponding colors) and of the baseline time-points pooled from all 10 subjects together (bold red line).

Note that the slopes of the subject-specific regressions (both linear and log-linear) are very different from the slopes of the population-specific regressions.
